# Crossed-Beams and Theoretical Studies of the Multichannel Reaction O(^3^P) + 1,2-Butadiene (Methylallene): Product Branching Fractions and Role of Intersystem Crossing

**DOI:** 10.1021/acs.jpca.5c03937

**Published:** 2025-08-26

**Authors:** Gianmarco Vanuzzo, Andrea Giustini, Adriana Caracciolo, Silvia Tanteri, Domenico Stranges, Marzio Rosi, Piergiorgio Casavecchia, Nadia Balucani, Maristella Di Teodoro, Sarah Nicole Elliott, Carlo Cavallotti

**Affiliations:** † Dipartimento di Chimica, Biologia e Biotecnologie, 9309 Università degli Studi di Perugia, 06123 Perugia, Italy; ‡ Dipartimento di Ingegneria Civile e Ambientale, Università degli Studi di Perugia, 06125 Perugia, Italy; § Dipartimento di Chimica, Università degli Studi La Sapienza, 00185 Roma, Italy; ∥ Computational Laboratory for Hybrid/Organic Photovoltaics (CLHYO), Istituto CNR di Scienze e Tecnologie Chimiche “Giulio Natta” (CNR-SCITEC), 06123 Perugia, Italy; ⊥ Dipartimento di Chimica, Materiali, Ingegneria Chimica “Giulio Natta”, 18981 Politecnico Milano, 20131 Milano, Italy

## Abstract

The reactions of ground state oxygen atoms, O­(^3^P), with unsaturated hydrocarbons (UHs) are relevant in the oxidation in different environments. They are usually multichannel reactions that exhibit a variety of competing product channels, some of which occur adiabatically on the entrance triplet potential energy surface (PES), while others occur nonadiabatically on the singlet PES that can be accessed via *intersystem crossing* (ISC). ISC plays a key role on the mechanism of these reactions, impacting greatly the product yields. Identification of all primary reaction products, determination of their branching fractions (BFs), and assessment of the role of ISC is central for understanding the mechanism of these reactions. This goal can be best achieved combining crossed-molecular-beam (CMB) experiments with universal, *soft* ionization, mass-spectrometric detection and time-of-flight analysis to high-level *ab initio* electronic structure calculations of triplet/singlet PESs and Rice-Ramsperger-Kassel-Marcus/Master Equation (RRKM/ME) computations of product BFs with inclusion of ISC effects. Over the years this combined approach was found to be rewarding and successful for O­(^3^P) reactions with the simplest alkynes, alkenes, and dienes containing two, three, or four carbon atoms. Here, we report the full experimental and theoretical work on the reaction O­(^3^P) + 1,2-butadiene that permits us to explore how the mechanism and product distribution vary when moving from O­(^3^P) + allene (propadiene) to O­(^3^P) + methylallene (1,2-butadiene) and when comparing this system to related C4 unsaturated systems, namely O­(^3^P) + 1-butene and O­(^3^P) + 1,3-butadiene. In the present CMB experiments at the collision energy of 41.8 kJ/mol we have observed and characterized nine different product channels. Synergistic *ab initio* transition-state theory-based master equation simulations coupled with nonadiabatic transition-state theory on the coupled triplet/singlet PESs were used for computing the product BFs and assisting the interpretation of the experimental results. Theoretical predictions and experimental results were found to be in overall good agreement. The finding of this work can be useful for the kinetic modeling of the oxidation of 1,2-butadiene and of systems involving 1,2-butadiene as an important intermediate.

## Graphical abstract

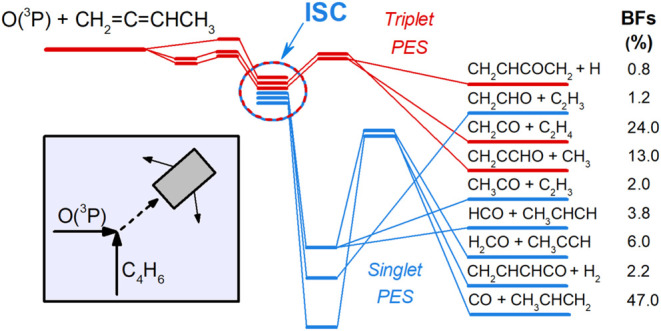

## 1 Introduction

The reactions of ground state oxygen atoms, O­(^3^P), with unsaturated hydrocarbons (UHs) and aromatic hydrocarbons (AHs) are of considerable relevance in areas ranging from combustion^1–7^ to plasma discharges^8^ and the Earth’s atmosphere,^9^ and play a role also in oxygen-rich interstellar and circumstellar environments.^10–12^ These reactions play a direct or indirect role in soot and PAHs formation.^13–15^ Among the UHs indirectly involved in the generation of these species, the butadiene (C_4_H_6_) isomers are well-known for their reactivity with atomic and molecular radicals participating in atmospheric chemistry. For instance, although 1,2-butadiene (CH_2_=C=CH–CH_3_) is not released by human activities as such, this unsaturated hydrocarbon was found to be involved in the isomerization mechanism of 1,3-butadiene (CH_2_=CH–CH=CH_2_) (*T* > 1500 K), which was recognized as a hazardous and carcinogenic, abundant air pollutant.^16–19^ Because of this, the photodissociation of 1,2-butadiene was investigated both experimentally^17,18^ and theoretically^19^ over the past decades Both pyrolysis experiments^17^ and laser photolysis molecular beam studies show that this UH mainly decomposes to CH_3_ + C_3_H_3_. In addition, this molecule was investigated for its reaction with more complex radical species involved in fuel combustion, such as the C_6_H_5_ (phenyl) radical, in synergistic molecular beam/theoretical investigations.^20^

Recent comparative studies of combustion characteristics of isomers, such as propanols,^21^ esters,^22^ di-isobutylenes,^23,24^ alkene^25^ isomers and, more recently, 1,3-butadiene and 1,2-butadiene isomers,^26^ have provided kinetic insights that turned out helpful in the construction and development of improved kinetic models.^26^ In fact, for hydrocarbons with the same structure but with different double bond positions, significant variance have been demonstrated^21–26^ in the ignition delays, the distribution of products in the oxidation, and in the laminar burning velocities. However, while there has been extensive work on 1,3-butadiene oxidation,^27^ much less exist on 1,2-butadiene oxidation.

In general, it is of fundamental and practical interest to investigate the effect of isomerism on chemical reactivity (for instance, 1,2-butadiene versus 1,3-butadiene), and also the effect of substituting in allene an H atom with a methyl group (methylallene versus allene). In the present combined experimental/theoretical study on the reaction dynamics of O­(^3^P) + 1,2-butadiene we are tackling these interesting issues. Specifically, after the early studies on O­(^3^P) reactions with 2C and 3C alkenes^28–34^ and dienes,^35^ the extension to the next members of the series, those containing 4C atoms, namely 1-butene^36^ and 1,3-butadiene^28^ (the simplest conjugated dienes) has permitted us to explore how the increasing molecular complexity affects the product distributions, branching fractions, and ISC.^37^ With the present study on O­(^3^P) + 1,2-butadiene (methylallene) we expand further this scrutiny by exploring how the reaction dynamics of this 4C diene varies with respect to the prototype diene, allene, and also with respect to the corresponding 4C alkene, 1-butene, and the simplest conjugated diene, 1,3-butadiene.

It is well-known that most bimolecular reactions of atomic or molecular radicals with polyatomic molecules, such as the reactions of O­(^3^P) with UHs and AHs, exhibit a large variety of primary product channels.^34,37^ From the reaction mechanism point of view, a common feature of these reactions is the initial formation (following the electrophilic O atom addition to the unsaturated bond(s)), of chemically activated triplet oxy-intermediate(s), that can evolve to products both adiabatically on the triplet potential energy surface (PES) and/or nonadiabatically (via Intersystem Crossing, ISC) on the low-lying singlet PES. Both triplet and singlet intermediates can evolve, via bond-cleavage or various steps of isomerization followed by bond-cleavage, to a variety of molecular/radical product channels. Knowledge of the primary product distribution (i.e., product branching fractions (BFs)) under combustion conditions for the reactions of UHs with O­(^3^P) is very valuable for the improvement of current combustion models. Notable examples are the reactions of O­(^3^P) with alkenes (ethylene,^29–31^ propene,^32,33^ 1-butene^36^), dienes (allene^35^), and conjugated dienes (1,3-butadiene^28^). Despite extensive kinetic and theoretical studies on these reactions since the mid-1950s,^38,39^ little information existed until recently about the nature of the primary reaction products and their BFs. It is certainly desirable to know their nature, their formation dynamics, the detailed reaction mechanism, and ultimately to be able to predict the channel-specific rate constants as a function of temperature and pressures, under conditions typical of combustion environments. However, this goal has represented a tremendous challenge, since the mid-1950s, for both experiment and theory. Experimentally, a technique able to probe, on the same footing, the different primary products of multichannel nonadiabatic reactions, such as those of O­(^3^P) with UHs, has to be *universal*. This stringent requirement, both in kinetic and dynamic experiments, confines the detection technique mainly to mass spectrometry, either with *soft* electron-impact ionization or *soft* photoionization.^34^ In fact, the well-known complication in mass spectrometry of dissociative ionization processes, require the use of *soft* ionization to suppress, or at least mitigate, the interferences in reactive signal detection arising from dissociative ionization processes of reactants, products, and background gases. This long-standing problem was successfully tackled only in the late 1990s/early 2000s with the implementation of *soft ionization* by tunable VUV radiation from third generation synchrotrons,^40–49^ both in kinetic^50,51^ and crossed molecular beam (CMB) experiments,^41–43,47–49^ and especially with the implementation of *soft* ionization by tunable low-energy electrons in CMB reactive scattering experiments.^34,52–54^

Over the past two decades in our laboratory we have elucidated the mechanism and dynamics of numerous multichannel nonadiabatic reactions of O­(^3^P) with 2C (ethylene,^29–31,53^ acetylene^52,55^ ), 3C (propene,^32,33^ propyne,^56,57^ allene^35^), and 4C (1-butene,^36^ 1,3-butadiene^28^) UHs, and with AHs (benzene,^58,59^ toluene^60^), heterocycles (pyridine^61^), and nitriles (cyanoacetylene,^62^ cyanoethylene^63^) by synergistically combining CMB scattering experiments with theoretical calculations of the underlying triplet and singlet PESs and their coupling, and statistical calculations of product branching fractions (BFs) taking into account ISC. One of the main aims has been the search for a relation between reactivity and molecular structure.^34,37^

Here, building on our previous investigations on O­(^3^P) + UHs, in particular on the O­(^3^P) + allene,^35^ O­(^3^P) + 1-butene,^36^ and O­(^3^P) + 1,3-butadiene^28^ reactions, we focus our attention on the oxidation of the next cumulene in the series, namely 1,2-butadiene (CH_2_=C=CH–CH_3_, methylallene).

The reaction of O­(^3^P) with 1,2-butadiene was investigated in 1986 in a kinetic experiment by Collin and Deslauries.^64^ Atomic oxygen was produced by O_2_ photolysis at 147 nm. In that work, the global rate constant at room temperature was determined (*k*_293K_ = 5.94 × 10^–12^ cm^3^ molecule^–1^ s^–1^); it was suggested that the main reactive channel was CO + C_3_H_6_ (propene). From final product analysis a variety of species were observed (crotonaldehyde, metacryl aldehyde, buten-2-one) that likely were reaction intermediates stabilized in the multiple-collision conditions of the flow experiments. No further experimental and/or theoretical investigations were reported on the title reaction since. In particular, for the bimolecular reaction O­(^3^P) + 1,2-butadiene the identity and the branching fractions of the primary products remained unknown until our recent CMB study of which we presented a preliminary report on the two main product channels observed and preliminary theoretical calculations of the underlying triplet PES (with only some minima values of the singlet PES).^65^

In the present work, we report the full account of the combined experimental and theoretical study of the O­(^3^P) + 1,2-butadiene reaction. Specifically, we detail (i) the measurements of product angular and time-of-flight (TOF) distributions as obtained in CMB experiments at the collision energy of 41.8 kJ/mol and the determination of the product BFs, (ii) the results of extended, high-level electronic structure calculations of the triplet and singlet PESs with inclusion of ISC between the triplet and singlet PESs, and (iii) the results of statistical RRKM/ME (Rice-Ramsperger-Kassel-Marcus/Master Equation) calculations of the product BFs on the coupled triplet/singlet PESs (i.e., with inclusion of ISC) for comparison with the experimental BFs. Using this synergistic experimental/theoretical approach we have identified theoretically a large variety (up to 12) of isomeric exothermic product channels (for a total of 32 isomeric channels) leading to H-displacement and H_2_ elimination channels, to CO (carbon monoxide) and CH_3_ (methyl) elimination, and to H_2_CO (formaldehyde), HCCO (ketenyl), HCO (formyl), CH_3_CO (acetyl), CH_2_CHO (vinoxy), CH_3_CHO (acetaldehyde), and CH_2_CO (ketene) formation channels, as well as H abstraction forming OH (hydroxyl), some occurring on the triplet PES and others on the singlet PES (via ISC). We have theoretically differentiated the various isomeric coproducts of the above primary products (for a total of 32 isomeric channels) and determined whether they occur adiabatically (**ad**) on the entrance triplet PES or nonadiabatically (via **ISC**) on the singlet PES. The 32 exothermic isomeric product channels of the title reaction are listed in Figure 1; they will be detailed in the Sections 4 and 5.

**Figure 1:**
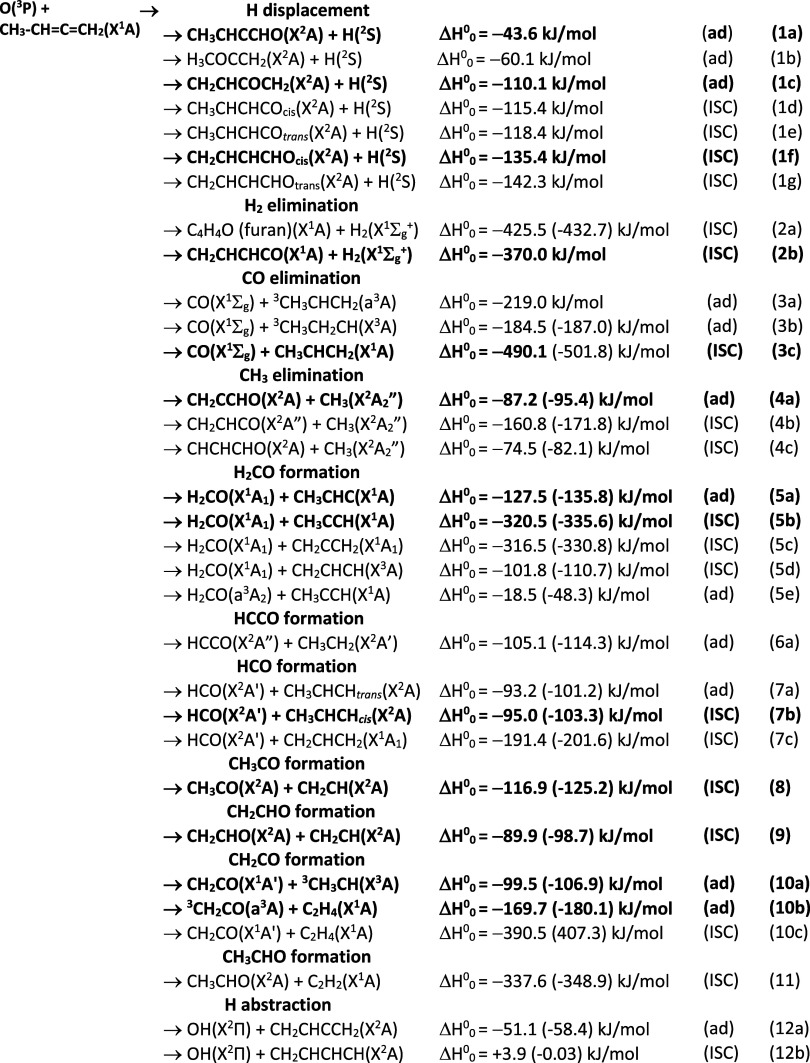
List of the 32 energetically allowed isomeric product channels of the O­(^3^P) + 1,2-butadiene reaction.

The reaction enthalpies for the 32 isomeric product channels (1–12) (see Figure 1) are those from the present electronic structure calculations (see Sections 3 and 5). The electronic state of reactants and products are indicated. Reaction enthalpy values agree, within the uncertainties, with literature values^66^ (values in parentheses) when these are available.

We have obtained experimental evidence of 9 channels among the 12 competing, isomeric product channels; the 9 characterized product channels are those reported in bold in Figure 1. As mentioned above they are those leading to formation of H, H_2_, CO, CH_3_, H_2_CO, HCO, CH_3_CO, CH_2_CHO, and CH_2_CO, and for these channels we have elucidated the reaction dynamics and derived the BFs. The results obtained on the O­(^3^P) + 1,2-butadiene (methylallene) reaction are compared with those obtained recently on the related reactions O­(^3^P) + allene,^35^ O­(^3^P) + 1-butene,^36^ and O­(^3^P) + 1,3-butadiene.^28^ A notable difference with respect to allene (CH_2_=C=CH_2_), 1-butene (CH_2_=CH–CH_2_–CH_3_), and 1,3-butadiene (CH_2_=CH–CH=CH_2_) is that for methylallene (CH_2_=C=CH–CH_3_) there exist three different attack sites for the O atom (attack to C1, C2, and C3) (see Scheme 1) and this implies a more complex PES on one side and a significantly different product distribution on the other, especially in terms of relative importance of radical channels and molecular channels. We remind that molecular channels lead to chain termination while radical channels to chain propagation, and this may have significant impact in real combustion systems involving 1,2-butadiene.

**Scheme 1:**
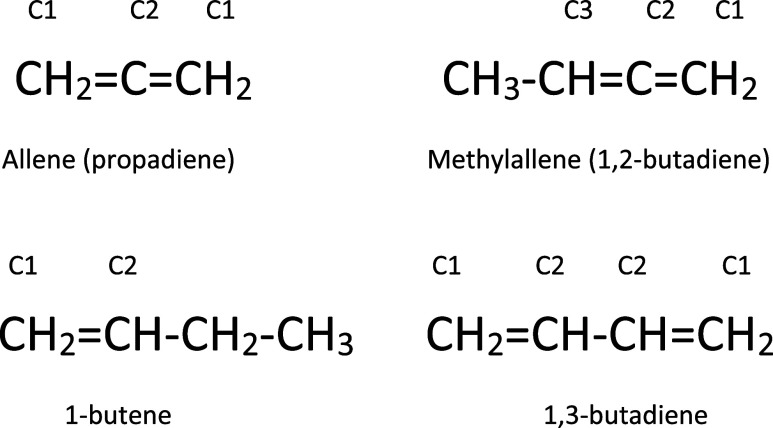
1,2-Butadiene Compared with the Simplest Diene, Allene, and with the Corresponding 4C Alkene, 1-Butene, and Conjugated Diene, 1-3-Butadiene^a^

a The numbering of the relevant C atoms involved in the unsaturated bonds are indicated with C1, C2, and C3.

We have organized the paper as follows. In the Sections 2 and 3 we describe the experimental and theoretical methods, respectively. In Section 4 we describe the triplet and singlet PESs that we have employed for interpreting the reactivity of the system and present the theoretical results of kinetic calculations as a function of temperature. In Section 5 the experimental results and their analysis are presented, while in Section 6 the results of the theoretical calculations of the product BFs are reported and compared with experimental BFs. A general discussion of the synergistic experimental and theoretical results is given in Section 6. Conclusions follows in Section 7.

## 2 Experimental Methods

The crossed molecular beam (CMB) apparatus employed in this study has been described in previous publications.^34,36,54^ Briefly, the CMB instrument features (i) a large scattering chamber maintained at 1.5 × 10^–6^ mbar during the experiments to ensure single-collision conditions (base pressure 2 × 10^–7^ mbar), (ii) two molecular beam sources positioned at 90°, (iii) a rotatable universal detector, based on an electron-impact (EI) ionization quadrupole mass-spectrometer (MS) housed in a triply differentially pumped chamber kept in ultrahigh-vacuum (UHV) conditions (≤1 × 10^–10^ mbar in the ionization region), and (iv) a time-of-flight (TOF) analysis system.

Collimated continuous supersonic beams of the two reactants are crossed at 90° in the scattering chamber. The MS detector, composed of a *tunable* EI ionizer followed by the quadrupole mass filter and a Daly type^67^ ion detector, can be rotated in the plane defined by the two reactant beams.^34,54^ Characteristics of the two supersonic reactant beams have been given in the previous partial report on this system.^65^ Here, we summarize only the main aspects relevant to the present full report on the combined CMB reactive scattering and theoretical study of the O­(^3^P) + 1,2-butadiene reaction. The supersonic beam of oxygen atoms seeded in helium, produced using a radio frequency (RF) discharge beam source,^68^ has a peak velocity of 2470 m/s and speed ratio of 4.9.^65^ The supersonic beam of pure 1,2-butadiene has peak velocity and speed ratio of 685 m/s and 4.4, respectively.^65^ Under the experimental conditions, the corresponding average collision energy, *E*_c_, was 41.8 kJ/mol and the center-of-mass (CM) angle 44.0° (from the O atom direction).

We recall that the atomic oxygen beam is composed mainly of ground-state atoms, O­(^3^P) (≥90%), and a small amount of excited atoms, O­(^1^D) (≤10%).^68^ It should be noted that, given the large reaction cross section of the O­(^3^P) reaction at the experimental *E*_c_ because of the submerged barriers for C1 and C2 addition, and very small barrier for C3 addition (see Section 4.1), the O­(^3^P) reaction contribution to the reactive signal is expected to be largely dominant with respect to the O­(^1^D) contribution. This is supported by the finding, during the data analysis, that the maximum values of the best-fit *P*(*E*′_T_) distributions for the various channels fall well within the total available energy for the O­(^3^P) reactions. We remind that the O­(^1^D) electronic energy is 190 kJ/mol with respect to the ground state.

The angular distribution of the various products, *N*(Θ), (i.e., the number density as a function of the LAB scattering angle Θ), is obtained by modulating the secondary (hydrocarbon) beam at a fixed frequency (160 Hz) for background subtraction and taking at least four scans per angle, using counting time of 50–100 s per angle at each scan. A chopper-wheel can be positioned in front of the entrance of the detector and used to measure the TOF distributions of the reactants (using the single-shot TOF method) and of the products at selected LAB angles (using the pseudorandom method). The TOF distributions of the various products, *N*(Θ,*t*), are measured at a number of selected LAB angles using the pseudorandom chopping technique with a wheel featuring four identical pseudorandom sequences of 127 open/closed slits. A dwell time of 6 μs/channel is obtained spinning the pseudorandom wheel at 328.1 Hz. TOF spectra were accumulated (typically for 3 to 5 h for each spectrum) at the center-of-mass angle (Θ_CM_ = 44°) for the mass-to-charge ratio *m*/*z* = 68, 43, 42, 41, 30, 29, 28, 27, and 15. In addition, we registered TOF spectra at 28° (forward direction with respect to O­(^3^P)) at *m*/*z* = 68, 43, 41, 29, 27, and 15, again with a counting time ranging from 3 to 5 h depending on the signal intensity and product mass.

The reactive scattering measurements were carried out in the LAB reference frame, while for the physical interpretation of the scattering process it is necessary to transform the LAB data to a reference frame that moves with the CM frame.^69,70^ To carry out the LAB to CM coordinate transformation we recall that the relation between the LAB and CM product fluxes, *I*_LAB_ and *I*_CM_, is given by *I*_LAB_(Θ,*v*) = *I*_CM_(θ,*u*)*v* ^2^/*u* ^2^, where Θ and *v* are the LAB scattering angle and velocity, respectively, while θ and *u* are the corresponding CM quantities.^69,70^ Since the EI-MS detector measures the number density of products, *N*(Θ), rather than the flux *I*_LAB_(Θ,*v*), the actual relation between the LAB number density *N*_LAB_(Θ,*v*) and the CM flux *I*_CM_(θ,*u*) is given by *N*_LAB_(Θ,*v*) = *I*_CM_(θ,*u*)*v*/*u* ^2^.^34,69,70^ The outcome of a reactive scattering experiment is the so-called double differential cross section *I*_CM_(θ,*u*), which is commonly factorized into the product of the CM velocity distribution *P*(*u*) (or translational energy distribution *P*(*E*′_T_)), and the CM angular distribution *T*(θ), that is *I*_CM_(θ,*E*′_T_) = *T*(θ)*P*(*E*′_T_). Because of the finite resolution of the experimental conditions (angular and velocity spread of the reactant beams and angular resolution of the detector), the LAB to CM transformation is not single-valued and, therefore, analysis of the LAB data is usually performed by forward-convoluting tentative CM angular and translational energy distributions (*T*(θ) and *P*(*E*′_T_), respectively) that are averaged and transformed into the LAB frame for comparison with the experimental distributions, and the procedure is repeated until the best-fit of the experimental distributions is obtained.^34^ In the double differential cross section in terms of *T*(θ) and *P*(*E*′_T_), the *T*(θ) and *P*(*E*′_T_) functions contain all the information about the reaction dynamics. When multiple reaction channels contribute to the signal at a given *m*/*z* ratio, a more complex situation arises (see ref 34). In these cases a weighted total CM differential cross section, that reflects the contributions of the various contributing channels, is used in the data analysis of the LAB distributions for a specific *m*/*z*, that is, *I*_CM_(θ,*E*′_T_)_total_ = ∑_*i*_ *w*_*i*_ × [*T*(θ) × *P*(*E*′_T_)]_*i*_. In the latter relation the parameter *w*_*i*_ represent the relative contribution of the integral reactive cross section of the *i*th reaction channel. Starting from the apparent cross sections of the various channels (i.e., the weights *w_i_* in the above equation) and following the procedure initially implemented by Schmoltner et al.,^71^ it is possible to determine the ratios between the yields of the various product channels and then the product branching fractions (BFs), as we have outlined elsewhere.^34^

## 3 Theoretical Methods

The potential energy surfaces for the O­(^3^P) + 1,2-butadiene system have been investigated by optimizing the geometries of critical points as local minima and first order saddle points using the ωB97X-D functional^72^ and the correlation-consistent valence-polarized aug-cc-pVTZ basis set,^73^ with the exception of the entrance channels and minimum energy crossing points (MECPs), as discussed below. Hessians were determined at the same level of theory and used to compute vibrational frequencies and zero-point energy corrections. Intrinsic reaction coordinate (IRC)^74,75^ calculations were performed for reactions proceeding through saddle points to ensure that they are connected to the desired reactants and products. The energies of all the critical points were then determined at the CCSD­(T)/aug-cc-pVTZ level^76,77^ on ωB97X-D structures. The CCSD­(T) single-point energies were corrected with zero-point energies computed at the ωB97X-D level of theory.

Because of the multireference nature of the wave functions, the energies of the saddle points of the three entrance channels were determined at the CASPT2/aug-cc-pVTZ level. The adopted (8e,7o) active space consisted of the (4e,3o) p orbitals of oxygen and of the (4e,4o) π and π* bonding and antibonding orbitals of 1,2-butadiene. Structures and Hessians were determined both at the ωB97X-D/aug-cc-pVTZ and CASPT2/aug-cc-pVTZ levels, finding consistent results. This confirms, as we found in recent works^58–60^ that determining energy of saddle points with multireference character at the CASPT2 level on ωB97X-D geometries is a computationally efficient and reasonably accurate approach. Simulations were performed using energies determined on CASPT2 structures.

The structures of MECPs between the triplet and singlet PESs were determined at the ωB97X-D/6–311+(g,p) level using the Lagrangian multiplier optimization method implemented in EStokTP.^78^ Bordered Hessians were computed at the same level of theory and harmonic frequencies were determined through its diagonalization following projection of translation, rotation, and motion through the crossing seam. Energies were computed at the CASPT2/aug-cc-pVTZ level using a (6e,5o) active space, consisting of the π and π* (2e,2o) orbitals, of the two singly occupied orbitals (2e,2o), and of the oxygen lone pair (2e,1o). Spin–orbit coupling coefficients between the triplet and singlet PES were computed at the MECP using a Breit–Pauli Hamiltonian and a CASSCF­(6e,5o)/cc-pVDZ wave function.

Canonical and microcanonical rate constants were computed using variational transition state theory (TST) for the entrance and H abstraction channels and conventional TST for isomerization reactions. The density of states of wells and saddle points was determined in the RRHO approximation, describing internal torsions using the 1D hindered rotor approximation when relevant. Hessians for the entrance channels were determined at the ωB97X-D/aug-cc-pVTZ both at the saddle point and along the minimum energy path (MEP), determined through IRC calculations. Vibrational frequencies were computed in internal coordinates along the MEP. The rate of ISC was computed using nonadiabatic microcanonical TST,^79^ estimating ISC probabilities with Landau–Zener theory.^80^ The energy barrier for H abstraction from the methyl group of 1,2-butadiene was also determined at the CASPT2/aug-cc-pVTZ level using a (10e,9o) active space averaged over two states on ωB97X-D/aug-cc-pVTZ geometries.

Branching fractions (BF) were determined performing stochastic master equation simulations using the KMC-RRKM code.^81^ Rate constants were computed microcanonically as a function of energy *E* and momentum *J*, with steps of 1 cm^–1^ up to a threshold of 100,000 cm^–1^, averaged over *J* as suggested by Miller at al.,^82^ and then averaged and discretized in bins of 100 cm^–1^ before performing the stochastic simulations. Simulations were performed at 10^–9^ bar, a condition in which collisions are absent for the considered number of reactive events. A total of 10000 reactive events were considered in each simulation to investigate the reactivity on the portions of the PES accessed through the three addition reactions of O­(^3^P) to the C1 (terminal), C2 (central), and C3 (methylene carbon atom) sites. Total branching fractions were determined weighting the number of reactive events calculated from each simulation by the addition probability, determined as the ratio between the channel-specific rate constant calculated at 300 K and the total addition rate constant, as done in our previous studies.^28,33,58–61^ The reason why rate constants for the entrance channels were computed at 300 K is that this is the thermal vibrational energy of the reactants, which is not dissipated despite the supersonic expansion as vibrational relaxation is expected to be minor, while it was assumed that the translational energy of the beam is not thermalized among the reactants because of insufficient coupling. According to our experience in simulating O­(^3^P) addition to unsaturated hydrocarbons, these assumptions allow to describe properly the branching among the possible addition sites.

All the ωB97X-D structure optimizations were carried out with the GAUSSIAN 09 program package^83^ while CASSCF, CASPT2, and CCSD­(T) calculations were performed with MOLPRO.^84^

## 4 Experimental Results and Analysis

### 4.1 Product Angular and TOF Distributions in the LAB Frame

The Newton (velocity vector) diagram, shown in Figure 2, depicts the kinematics of the bimolecular reaction of O­(^3^P) with 1,2-butadiene at *E*_c_ = 41.8 kJ/mol, and illustrates how the possible reaction products can be scattered in angle and velocity with respect to the center-of-mass (CM) of the system.^69,70^ In the Newton diagram each superimposed circle is related to a primary product detected in the experiment (the observed product is specified, while the coproduct is indicated within parentheses). Each circle is drawn by considering the maximum CM speed that each indicated product (the first one listed for each product channel) can attain if all the total available energy, *E*_TOT_, for that channel (*E*_TOT_ = *E*_c_ – Δ*H*_0_^0^) is converted into product translational energy. Note that the circles are different for the two coproducts of a given channel, being their corresponding mass different. The first indicated coproduct is the one detected experimentally. Note also that only for some channels both coproducts could be detected.

**Figure 2:**
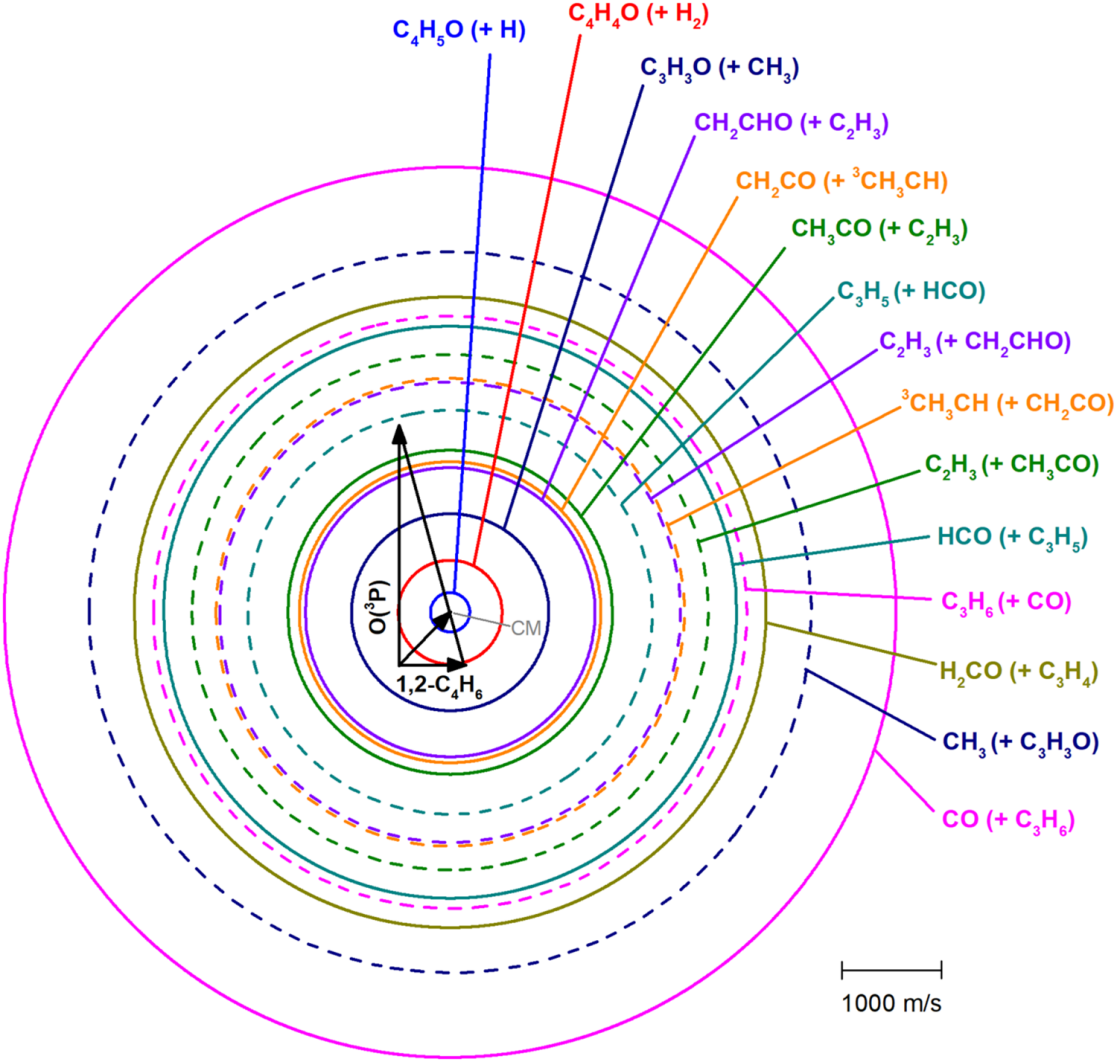
Newton (velocity vector) diagram for the O­(^3^P) + 1,2-butadiene reaction (*E*_c_ = 41.8 kJ/mol) with superimposed circles delimiting the maximum speed in the CM reference frame (the velocity vector of the CM is labeled, with Θ_CM_ = 44°) and the LAB scattering angular range of the various, indicated primary products. The velocity vectors of the two reactants species, which cross each other at 90° in the LAB frame, and the velocity vector of the CM of the system are indicated. Nine different reactive channels were identified: C_4_H_5_O + H (blue line), C_4_H_4_O + H_2_ (red line), CH_3_CO + C_2_H_3_ (green line), CH_2_CHO + C_2_H_3_, CH_2_CO + CHCH_3_ (orange line), CO + C_3_H_6_ (magenta), HCO + C_3_H_5_ (cyan line), H_2_CO + C_3_H_4_ (navy line) and C_3_H_3_O + CH_3_ (black line). The O-containing primary products are represented by solid lines, while the dashed lines correspond to the hydrocarbon coproducts. Circles of the two coproducts are depicted only for the channels of CO, CH_3_, HCO, and CH_2_CO formation. Continuous circles refer to oxygenated coproducts, while dashed circles to hydrocarbon molecule/radical coproducts.

The H-displacement channels (1) exhibit favorable kinematics; in fact, the detected C_4_H_5_O heavy coproduct(s) is (are) confined within a much smaller Newton circle (and then is (are) strongly enhanced in the LAB frame by a favorable CM → LAB Jacobian transformation^69,70^ ) because the H atom coproduct has not internal energy, compared to the circles associated with the comparatively lighter detected coproducts of the various C–C bond-breaking channels (3–12) left by molecular/radical products that carry instead, besides translational energy, also internal energy. Note that in Figure 2 the circle of the H channel refers to channel (1a) (in the scale of the figure the different isomeric channels (1) would be essentially indistinguishable). In the cases of channels (3–12), because two cofragments of comparable mass are produced, due to linear momentum conservation^69,70^ the corresponding products are scattered over much wider Newton circles and then over much larger ranges of LAB scattering angles than channels (1). The circle of the heavy coproduct of channel (2b) is substantially larger than that of channel (1a) because H_2_, despite having some internal energy, has a mass twice that of H.

Reactive signals were observed at the following *m*/*z* ratios: 69 (C_4_H_5_O^+^), 68 (C_4_H_4_O^+^), 67 (C_4_H_3_O^+^), 43 (CH_3_CO^+^), 42 (C_3_H_6_^+^, CH_2_CO^+^), 41 (C_3_H_5_^+^), 30 (H_2_CO^+^), 29 (HCO^+^), 28 (CO^+^), 27 (C_2_H_3_^+^), and 15 (CH_3_^+^), where the ions in parentheses refer to the parent ion of a specific primary product (*m*/*z* = 69, 68, 43, 42, 41, 30, 29, 28, 27, and 15), but often also to a daughter ion of other specific products. For instance, *m*/*z* = 68 is parent ion of the C_4_H_4_O product and daughter ion of the C_4_H_5_O product, *m*/*z* = 67 is instead daughter ion of both C_4_H_4_O and C_4_H_5_O products, *m*/*z* = 42 is both parent ion of CH_2_CO (ketene) and C_3_H_6_ (propene) but also daughter ion of CH_2_CHO (vinoxy), *m*/*z* = 29 is parent ion of HCO (formyl) but also daughter ion of H_2_CO (formaldehyde).

Concerning the product LAB angular distributions, measurements were carried out for all the above *m*/*z* ratios (see Figure 3), except *m*/*z* = 69, 67, 30, and 28, for which the S/N ratio was too low. On the other hand, the TOF spectra for all the observed *m*/*z* values (except for *m*/*z* = 69, 67, and 30 because of the poor S/N) were acquired at the CM angle (Θ_CM_ = 44°) (see Figure 4(*lhs*) for *m*/*z* = 68, 43, 42, and 41, and Figure 4(*rhs*) for *m*/*z* = 29, 28, 27, and 15). For these same masses, except *m*/*z* = 28, TOF spectra were also measured at Θ = 28° (forward direction with respect to the O atom) (see further below). The measurement at *m*/*z* = 68, corresponding to the heavy coproduct of the H displacement channel (1) and of the H_2_ elimination channel (2), were performed using *hard ionization* (70 eV electron energy), while for the other *m*/*z* values *soft ionization* (at 17 eV) was employed in order to suppress/mitigate the interferences related to dissociative ionization processes of the hydrocarbon reactant, products, and background gases. Furthermore, for *m*/*z* = 69, 68, and 67 intensities at the peak of the angular distribution were also acquired at 17 eV for normalization purposes. We recall that experimentally we cannot distinguish directly the different isomer products of channels (1–12). Notably, no reactive signal was observed at *m*/*z* = 17 (OH^+^) that indicates that the weakly exothermic OH forming channels (12) are negligible at the experimental *E*_c_.

**Figure 3:**
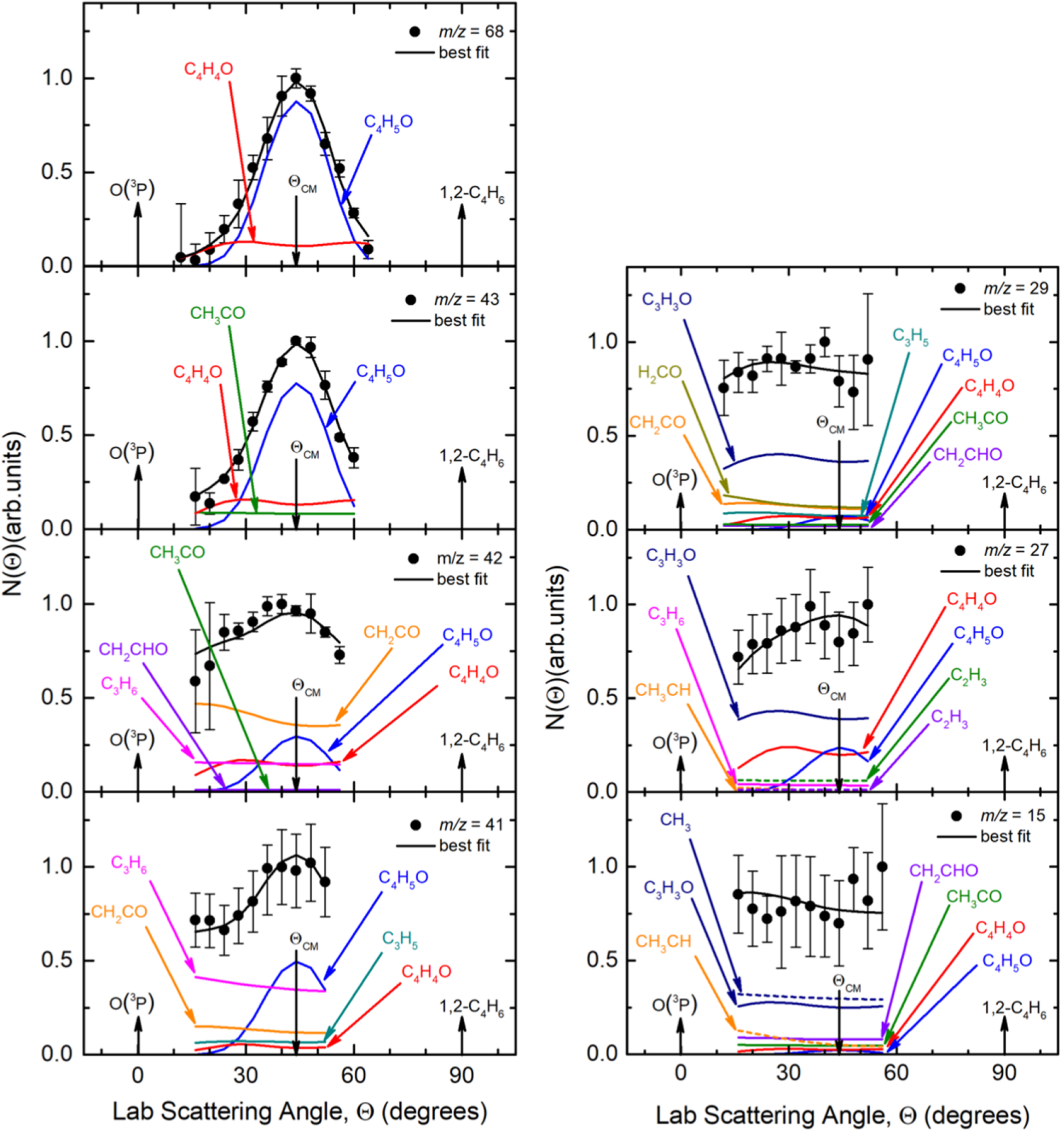
Product LAB angular distributions measured at *m*/*z* = 68, 43, 42, 41­(*lhs*) and 30, 29, 27, and 15 (*rhs*) for the O­(^3^P) + 1,2- butadiene reaction at *E*_c_ = 41.8 kJ/mol. The black solid curve superimposed on the experimental data (black dots) corresponds to the calculated global best-fit using the CM functions depicted in the Figures 12 and 13. The separate contributions to the calculated global LAB product angular distributions are color-coded and indicated with the formula of the corresponding product (color coding as in Figure 2).

**Figure 4:**
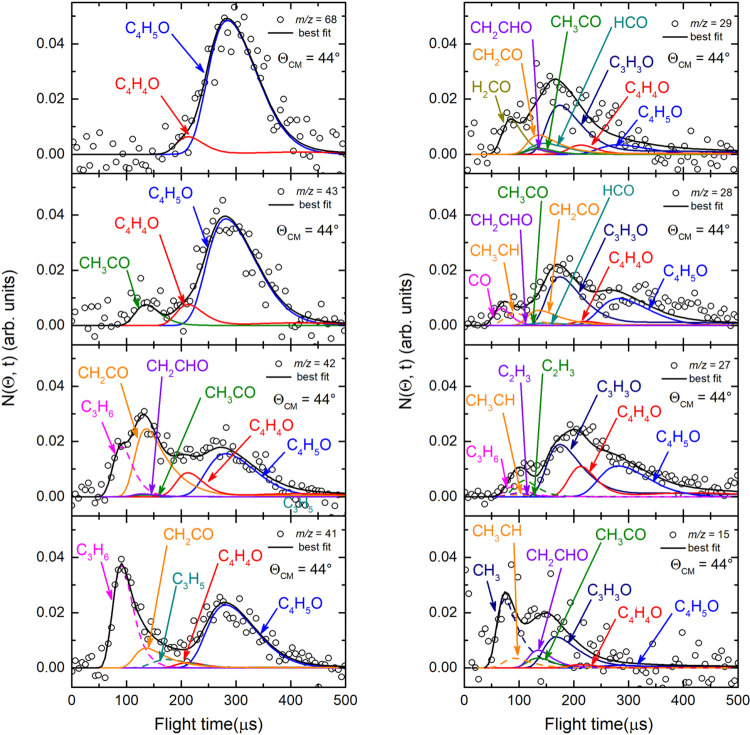
(*lhs*) TOF distributions measured at Θ_CM_ = 44° for *m*/*z* = 68 (70 eV) and *m*/*z* = 43, 42, and 41 exploiting *soft ionization* (17 eV). (*rhs*) TOF distributions measured for *m*/*z* = 29, 28, 27, and 15 at Θ_CM_ = 44° exploiting *soft ionization* (17 eV). In each panel the black solid curve superimposed on the experimental data (empty dots) corresponds to the calculated global best-fit using the CM functions depicted in Figures 12 and 13. The distinct contributions to the calculated global LAB TOF distributions are color-coded and indicated with the formula of the corresponding product (color coding as in Figure 2).

The heavy coproducts of the H-displacement channels (1) and the H_2_-elimination channels (2) were characterized by measurements at *m*/*z* = 68 (C_4_H_4_O^+^ that corresponds to the parent ion of C_4_H_4_O from channel (2) and the daughter ion of C_4_H_5_O from channel (1)), while at lower *m*/*z* values we have identified seven additional product channels originating from the rupture of C–C bonds. Specifically, at *m*/*z* = 43 we detected the acetyl (CH_3_CO) radical (channel (8)) via its parent ion mass (CH_3_CO^+^), along with the heavy coproducts of the H and H_2_ forming channels. It should be noted that the acetyl isomer, vinoxy (CH_2_CHO), is not detectable at its parent mass of 43 (CH_2_CHO^+^), even at 17 eV, because it is well-known that it fragments strongly to *m*/*z* = 42 (and also to *m*/*z* = 29 and 15).^54^ In fact, the occurrence of a small amount of the vinoxy channel was assessed at the daughter ions with *m*/*z* = 42, 29, and 15. The ketene (CH_2_CO) and propene (C_3_H_6_) forming channels were probed at the corresponding parent ion masses of 42 (CH_2_CO^+^ and C_3_H_6_^+^, respectively). We emphasize that formation of the propene product was also confirmed by an intense signal at its daughter ion C_3_H_5_^+^ (*m*/*z* = 41). We obtained information on the other product channels from measurements at *m*/*z* = 29, 28, 27, and 15 that allowed us to identify H_2_CO/HCO, CHCH_3_/C_2_H_4_/CO, and CH_3_ primary species, respectively (see Figures 3 and 4). We remark that despite the use of *soft ionization*, the dissociative ionization of some of the relevant product parent ions was not completely suppressed. In addition, measurements at *m*/*z* = 28 were somewhat problematic because of the very high inherent detector background at this mass. Nevertheless, despite the high background observed at this *m*/*z* value, we were able to register the TOF distribution for this mass at the CM angle (see Figure 4(*rhs*)) and observe the coproduct of C_3_H_6_ (channel (3c)). We now examine in more detail the reactive signals at the various *m*/*z* values.

### 4.2 The *m*/*z* = 68 Data: H Displacement and H_2_ Elimination Channels

The occurrence of the atomic hydrogen displacement channel and of the H_2_ elimination channel was investigated at *m*/*z* = 69 and 68. However, because the reactive signal at *m*/*z* = 69 was hidden by the elastic scattering due to some hydrocarbon beam impurities, the heavy coproducts related to H displacement and H_2_ elimination channels, namely C_4_H_5_O and C_4_H_4_O, respectively, were characterized from measurements at *m*/*z* = 68 parent ion of C_4_H_4_O (channels (2)) and daughter ion of C_4_H_5_O (channels (1)) for which the S/N ratio was best. Angular and TOF distributions at *m*/*z* = 68 permitted us to describe the dynamics of both channels, because they exhibit a different exothermicity, kinematics, and dynamics. In fact, because of linear momentum conservation, the coproduct of H_2_, namely C_4_H_4_O, scatters in a much wider angular range compared to C_4_H_5_O (the coproduct of H), which is left by the very light H atom having half of the mass of H_2_. Furthermore, the H_2_ elimination channel is much more exothermic than the H forming channel and is characterized by a much higher exit barrier on the PES, all factors that make the P­(E′_T_) more energetic. (i.e., peaking and dying off at much higher energies). If we examine the LAB angular distribution registered at *m*/*z* = 68 between 12° and 64° (see Figure 3(*lhs*)-top panel), we note that it is bell shaped and peaks at the center-of-mass angle (Θ_CM_ = 44°), indicating that at this mass the C_4_H_5_O coproduct of the H-displacement channel is dominant in the LAB frame. However, if we move away from Θ_CM_, we can notice that the contribution attributed to C_4_H_5_O is not sufficient to reach the intensity measured at angles significantly smaller and larger than Θ_CM_. This indicates that, in addition to the coproduct of the H channel, there is the contribution of another product that can only correspond, on the basis of conservation of linear momentum and energy, to the coproduct of the H_2_ channel, C_4_H_4_O, detected at the *m*/*z* parent ion mass. This hypothesis is supported by the TOF distributions measured at Θ = 28° and 44° for the same *m*/*z* value (see Figure 5). As can be seen in Figure 5 (bottom panel) the *m*/*z* = 68 TOF spectrum at Θ_CM_ = 44° shows an intense, slow peak related to C_4_H_5_O (from channel (1)), and a small fast shoulder attributed to C_4_H_4_O from channel (2); this attribution is clearly confirmed by the TOF spectrum at Θ = 28° (Figure 5-top panel), at which the reactive contributions in the LAB frame of the H and H_2_ channels become comparable (see the LAB angular distribution at *m*/*z* = 68 shown in Figure 3(*lhs*-top panel)) and hence more clearly distinguishable in time in the TOF data.

**Figure 5:**
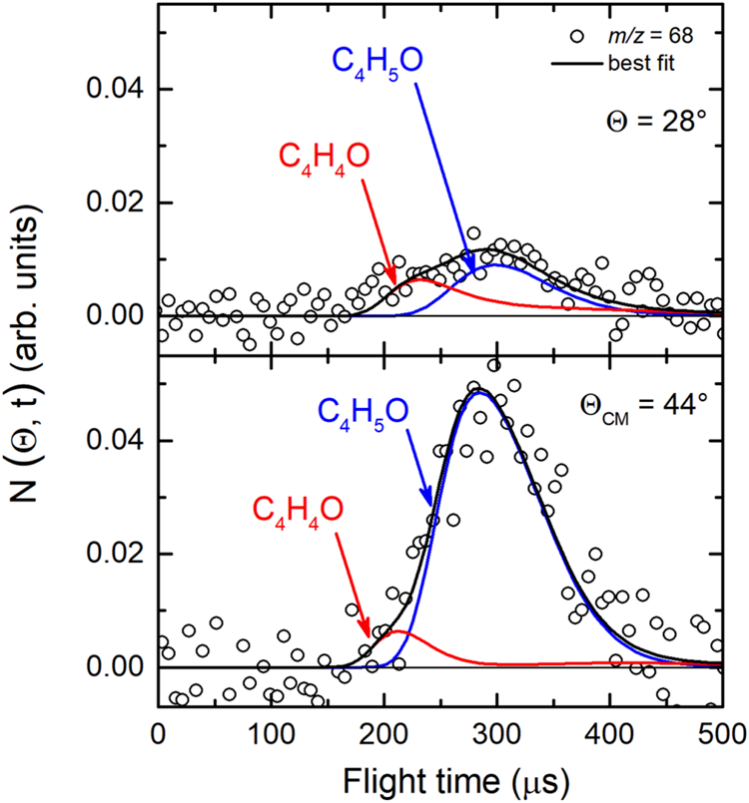
TOF distributions for *m*/*z* = 68 at two different angles (Θ = 28° and 44°) at 70 eV (*hard ionization*). The black solid curve represents the calculated global best-fit using the CM functions shown in the Figures 12 and 13. At this *m*/*z* value the broad slow peak is related to the heavy coproduct of the H channel 1 (blue), while the faster shoulder corresponds to C_4_H_4_O from the H_2_ elimination channel 2 (red).

We emphasize that the fragmentation of the heavy coproducts of the H and H_2_ forming channels is observed also at lower *m*/*z* ratios. In fact, both C_4_H_5_O and C_4_H_4_O are also detected at *m*/*z* = 43, 42, 41, 29, 28, and 27. In particular, if we examine the TOF spectra at these masses recorded at the CM angle (see Figure 4), we can notice that, for instance, the fragmentation of the C_4_H_5_O species is high at *m*/*z* = 43 as well as at *m*/*z* = 42 and 41, while it decreases at lower *m*/*z*, such as *m*/*z* = 28 and 27, and becomes almost negligible at *m*/*z* = 29 and 15 with respect to other contributing channels at those masses.

### 4.3 The *m*/*z* = 43, 42, and 41 Data: CH_3_CO + C_2_H_3_, CH_2_CHO + C_2_H_3_, CH_2_CO + C_2_H_4_, C_3_H_6_ + CO, and C_3_H_5_ + HCO Channels

After the analysis of the highest mass, where the dynamics of the H and H_2_ channels were characterized, we now focus on the primary products coming from C–C bond rupture processes of the relevant triplet and singlet reaction intermediates.

Product angular and TOF distributions at *m*/*z* = 43, 42, and 41 were measured using 17 eV electron energy. The angular distributions registered in the LAB angular range 16°–60°, 16°–56° and 16°–52°, for *m*/*z* = 43, 42, and 41, respectively, are depicted in Figure 3(*lhs*). Notably, as mentioned previously, although for these mass-to-charge ratios we exploited *soft ionization*, the contribution related to the fragmentation of the C_4_H_5_O and C_4_H_4_O species are still well observable in the TOF spectra (Figure 4(*lhs*)). We remark that the wider angular distributions recorded at *m*/*z* = 43, 42, and 41, with respect to that measured at *m*/*z* = 68, suggest that there are additional channel contributions from the title reaction, expectedly related to primary products originated from C–C bond breakage of the relevant intermediates. For instance, if we compare the angular distribution acquired at *m*/*z* = 68 with that registered at *m*/*z* = 43 (see Figure 3(*lhs*)-top two panels), although both are bell-shaped, at the lower *m*/*z* value we notice that the relative intensity in both backward and forward directions is somewhat higher (with respect to the peak of the distribution) than that registered at *m*/*z* = 68. This indicates that at *m*/*z* = 43 additional lighter products contribute, scattered over a wider Newton circle than those of C_4_H_5_O and C_4_H_4_O products. For the title reaction, the only possible product at this *m*/*z* value is the acetyl radical (CH_3_CO) (channel (8)), because the parent ion of its isomer product vinoxy (CH_2_CHO) (channel (9) is not stable, neither when using 17 eV electron energy.^54^ Consequently, in the LAB frame this new, lighter primary product is faster than C_4_H_5_O and C_4_H_4_O, as shown by the TOF spectra in Figure 4(*lhs*) at Θ_CM_ = 44° and in Figure 6 (at Θ_CM_ = 44° and Θ = 28°). The *m*/*z* = 43 TOF spectrum alludes, in fact, to the occurrence of the CH_3_CO + C_2_H_3_ reactive channel (8) through the detection of the acetyl radical at its parent ion mass (CH_3_CO^+^). Therefore, from the data at *m*/*z* = 43 we have assessed unambiguously the occurrence of, besides the H and H_2_ channels, also the CH_3_CO + C_2_H_3_ channel (8).

**Figure 6:**
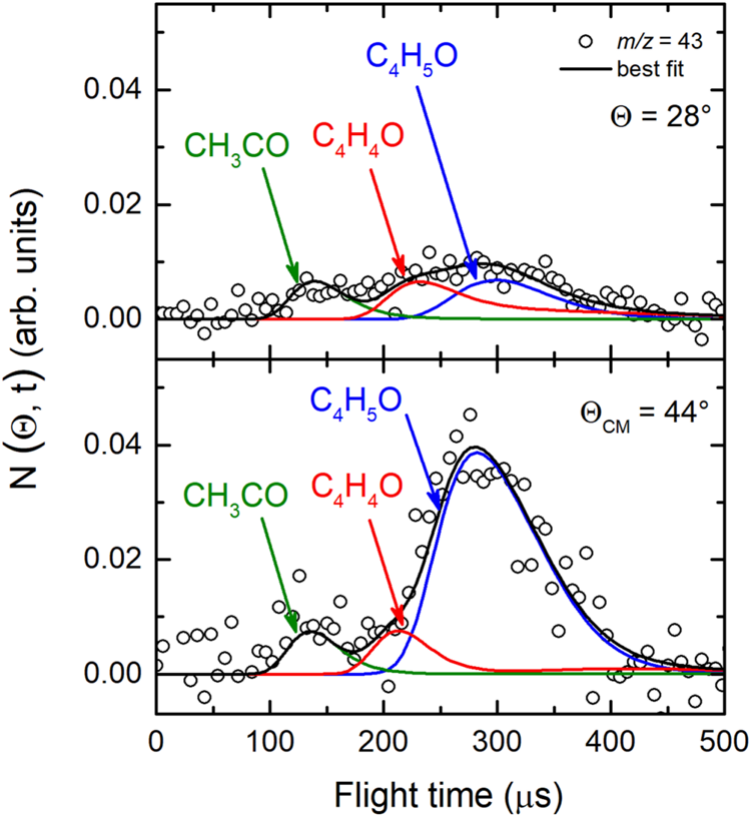
TOF distributions measured for *m*/*z* = 43 at Θ = 28° (corresponding to the forward direction) and Θ = 44° (corresponding to the CM) exploiting *soft ionization* (17 eV). The solid black curve represents the calculated best-fit by using the CM functions shown in the Figure 12). At this *m*/*z* value the slower peaks are due to the two products already characterized at *m*/*z* = 68, namely the heavy coproduct of the H channel (blue) and the H_2_ channel (red), while the faster peak is related unambiguously to acetyl formation (green).

Notably, the LAB angular distributions measured for both *m*/*z* = 42 and 41 are wider than those acquired at *m*/*z* = 68 and 43 (see Figure 3(*lhs*)). This indicates that at these *m*/*z* values new reactive channels can be identified for the title reaction. In fact, the TOF distributions registered at these *m*/*z* values are also very different with respect to those recorded at *m*/*z* = 68 and 43 (see Figure 4(*lhs*)). The introduction of the CH_2_CO (ketene) and C_3_H_6_ (propene) channels (3) and (10), respectively, was crucial in the data analysis for achieving the best-fit of the raw data at *m*/*z* = 42 and 41 (see Figures 3, 4, 7, and 8). Specifically, the ketene contribution is well visible at *m*/*z* = 42 (peaking at about 130 μs) and much less at *m*/*z* = 41 (see Figures 7 and 8, respectively). In contrast, the propene contribution appears as a fast shoulder on the ketene peak at the *m*/*z* = 42 parent ion and as a strong, dominant peak at *m*/*z* = 41 daughter ion. Notably, the TOF spectra acquired at *m*/*z* = 41 suggest that also a small amount of the HCO + C_3_H_5_ channel (7) is present, being *m*/*z* = 41 the parent ion of the propyl radical (C_3_H_5_) (see Figure 8). It should be noted that also very small amounts of vinoxy and acetyl are contributing to the *m*/*z* = 42 signal (Figure 7). This is corroborated by a comparatively more abundant contribution at *m*/*z* = 29 and 15 (see Figures 9 and 11) (see ref 54 and refs 29–31 for vinoxy detected at *m*/*z* = 29 and 15). Notably, at *m*/*z* = 41 channel (6) (HCCO formation) was not observed to occur and therefore it was considered negligible.

**Figure 7:**
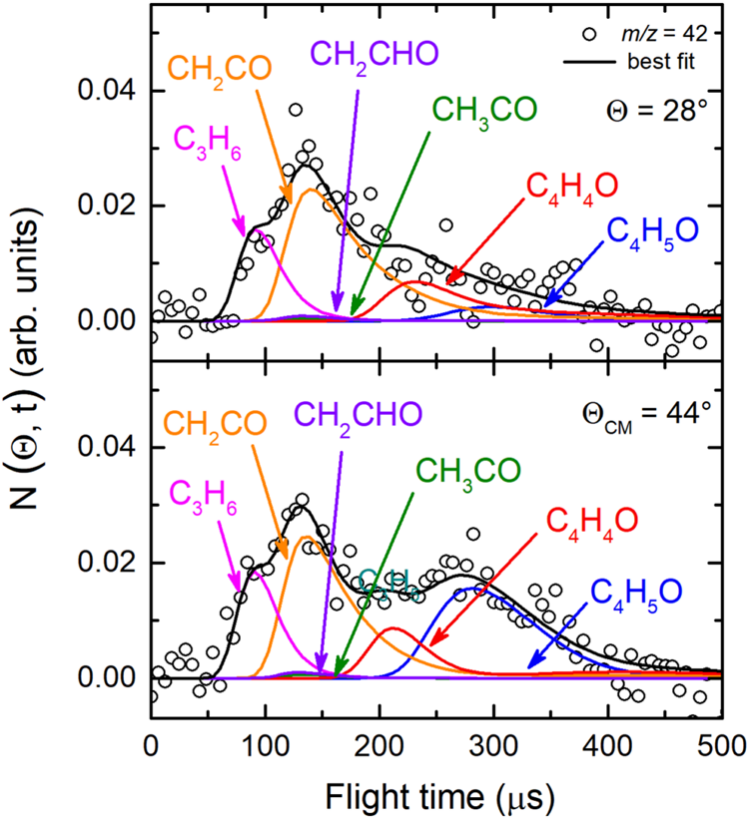
TOF distributions measured at *m*/*z* = 42 at two different angles (Θ = 28° and 44°) by exploiting *soft ionization* at 17 eV. The black solid curve indicates the calculated best-fit using the CM functions depicted in Figures 12 and 13. Besides the products characterized at *m*/*z* = 68 and 43, at this *m*/*z* value also propene (magenta), ketene (purple), and vinoxy products have been identified. The best-fit at this mass is a significant refinement with respect to that presented in Figure 3 of ref 65.

**Figure 8:**
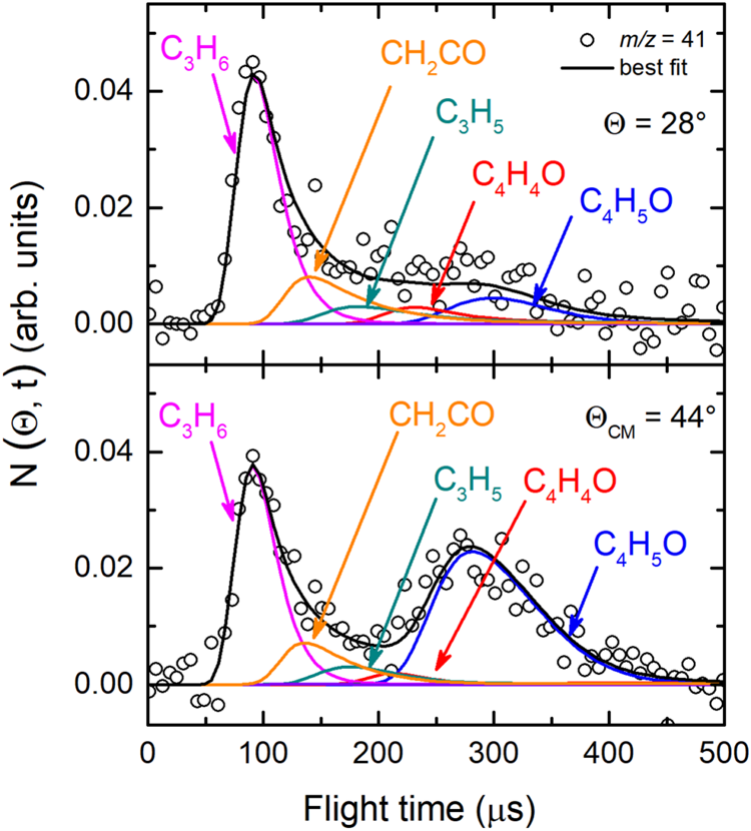
TOF distributions measured for *m*/*z* = 41 at Θ = 28° (forward direction) and Θ = 44° (CM angle) exploiting *soft ionization* at 17 eV. The black solid curve represents the calculated best-fit by using the best-fit CM functions shown in the Figures 12 and 13. At this *m*/*z* value, in addition to the reaction products characterized at *m*/*z* = 68, 43, and 42, the heavy coproduct C_3_H_5_ (allyl radical) (cyano line) of the HCO radical (channel (7)) was identified. Note that also this best-fit is a significant refinement with respect to that presented initially in Figure 4 of ref 65.

**Figure 9:**
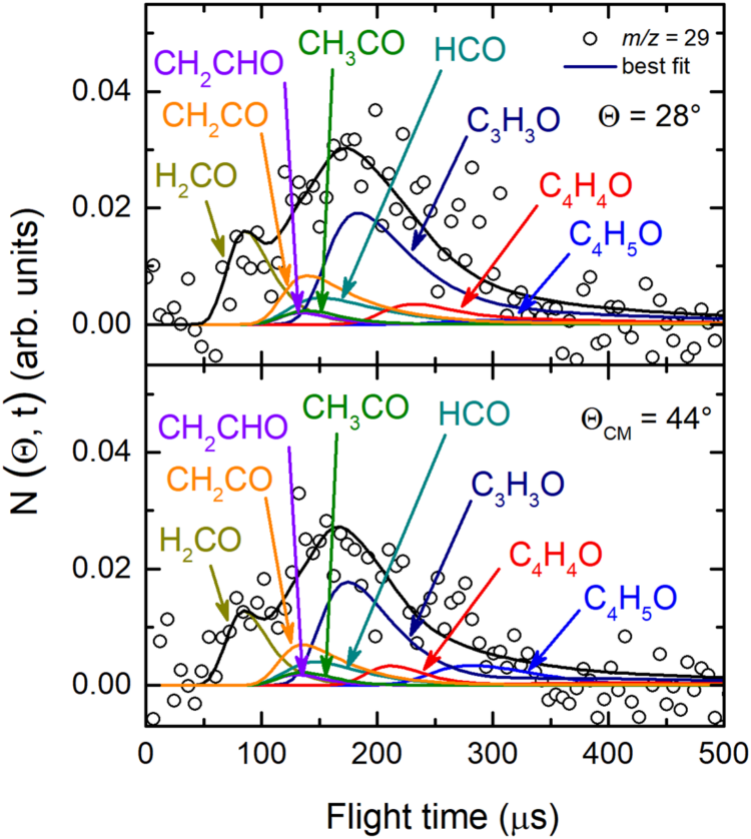
TOF distributions registered for *m*/*z* = 29 at Θ = 28° and Θ = 44° exploiting *soft ionization* at 17 eV. The black solid curve represents the calculated best-fit using the best-fit CM functions reported in the Figures 12 and 13. At this *m*/*z* value CHCH_3_ and HCO products are momentum matched to CH_2_CO and C_3_H_5_ (channels (10) and (7), respectively) that were characterized at *m*/*z* = 42 and 41, respectively (see Figures 7 and 8).

### 4.4 The *m*/*z* = 29, 28, 27, and 15 Data: H_2_CO + C_3_H_4_, HCO + C_3_H_5_, and CH_3_ + C_3_H_3_O Channels

The use of *soft ionization* at 17 eV was not sufficient to completely suppress the elastic signal deriving from the molecular beam of 1,2-butadiene (which at 17 eV still fragments to *m*/*z* = 29, 28, 27, and 15), thus producing an interference during the angular and TOF distribution measurements at these masses. This problem was overcome by acquiring the angular and velocity distributions at 17 eV also for the parent ion of the 1,2-butadiene reactant (*m*/*z* = 54). These data were then used for the correction of the angular distributions recorded at *m*/*z* = 29, 27, and 15 (see Figure 3(*rhs*)), and the TOF distributions recorded at *m*/*z* = 29, 28, 27, and 15 (see Figure 4(*rhs*)). The corrected data are those reported in the Figures 3, 4, 9, 10, and 11.

**Figure 10:**
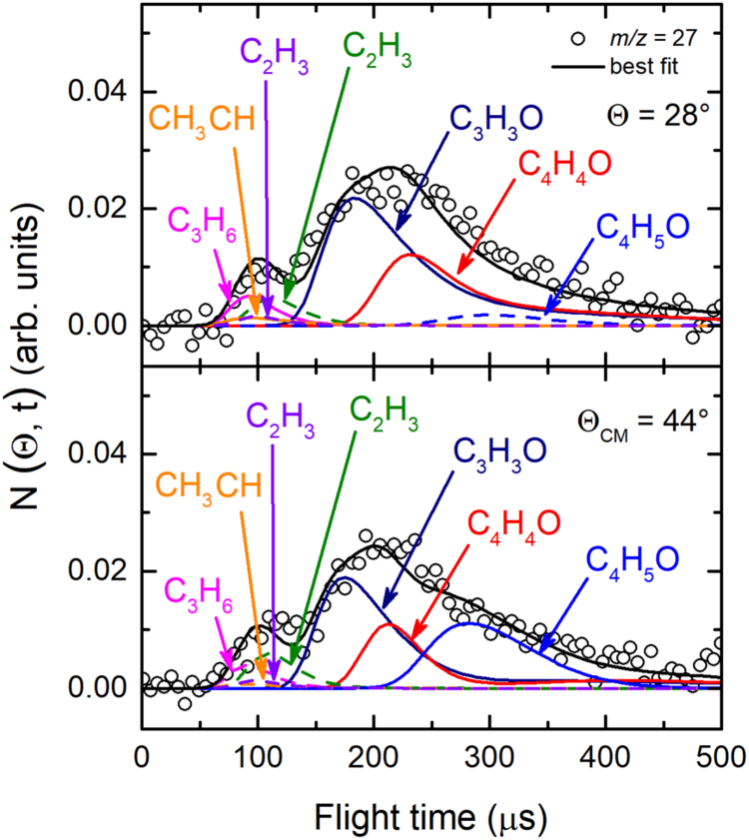
TOF distributions registered for *m*/*z* = 27 at Θ = 28° and Θ = 44° exploiting *soft ionization* at 17 eV. The black solid curve superimposed to the experimental data (empty dots) represents the calculated best-fit using the best-fit CM functions shown in Figures 12 and 13. The various contributions at this mass are indicated (color coding is as in the Figures 2–8).

**Figure 11:**
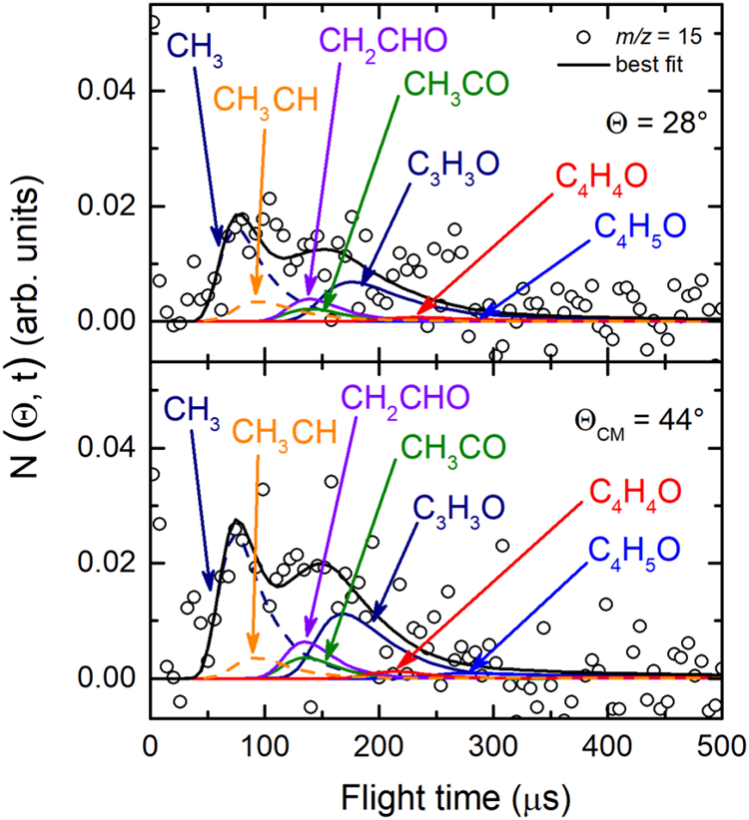
TOF distributions registered for *m*/*z* = 15 at Θ = 28° and Θ = 44° exploiting *soft ionization* at 17 eV. The black solid curve represents the calculated best-fit using the best-fit CM functions shown in the Figures 12 and 13. At this mass-to-charge ratio CH_3_ formation was identified via its parent ion CH_3_^+^, corroborating the identification of its heavy coproduct (C_3_H_3_O) at higher *m*/*z* values (see Figures 4(*rhs*), 9, and 10). Other contributions at this mass are also indicated (color coding as in Figure 2).

Regarding the data at *m*/*z* = 29 and 27, the product angular distributions were measured in the 12°–54° angular range, while for *m*/*z* = 15 the angular distribution was registered in the 14°–56° LAB range (see Figure 3(*rhs*)). At these *m*/*z* ratios the angular distributions are structureless and almost flat. This indicates the presence of new primary products that scatter into wider circles with respect to those species detected at higher *m*/*z* values. Therefore, to disentangle the primary products, analysis of the TOF spectra was crucial.

Figure 9 depicts the TOF spectra acquired for *m*/*z* = 29 at two LAB angles. In addition to a minor amount of the C_4_H_5_O and C_4_H_4_O contributions (from the H and H_2_ forming channels) (already characterized at *m*/*z* = 68), these spectra feature two faster contributions corresponding to H_2_CO (formaldehyde) (via its daughter ion HCO^+^) and C_3_H_3_O (via its daughter ion HCO^+^), where the latter can be identified as the heavy coproduct of the CH_3_ radical from channel (4). The TOF at *m*/*z* = 30 (not shown) had a low intensity and was very noisy; therefore, it could not be analyzed reliably; however, the TOF did feature a small fast component attributable to the H_2_CO parent ion, which was only 10–20% of the fast component at *m*/*z* = 29, an indication that the hot formaldehyde product fragments strongly at its daughter ion HCO^+^ (*m*/*z* = 29). Neglecting the small intensity of the H_2_CO parent ion leads to a slight underestimate of the formaldehyde channel BF that is well within the error bars (see Section 4.6 for the BFs). Regarding C_3_H_3_O (the coproduct of CH_3_), unfortunately the identification of this species via its parent ion at *m*/*z* = 55 was not possible because at this *m*/*z* value the reactive signal was very low, with most of the signal actually arising from the 5.5% (due to ^13^C natural abundance) of the signal due to elastically scattered 1,2-butadiene. However, the occurrence of the CH_3_ channels (4) is validated with the acquisition of the TOF spectra at *m*/*z* = 15 (see Figure 11), wherein the sharp fastest peak is unambiguously related to the CH_3_ product (via its parent ion) from channel (4), which is momentum matched with C_3_H_3_O, corroborating the substantial presence of the latter at *m*/*z* = 29 (see Figure 9), at *m*/*z* = 28 (see Figure 4-*rhs*), and *m*/*z* = 27 (see Figure 10). In the same way, we observed the momentum matching also for channels (8–12), by acquiring the TOF spectra besides at *m*/*z* = 29 (Figure 9), also at *m*/*z* = 28 (see Figure 4(*rhs*)-second panel from top) and *m*/*z* = 27 (Figure 10), identifying the coproducts of C_3_H_5_, CH_3_CO, CH_2_CHO, CH_2_CO, and C_3_H_6_ species, namely HCO (channels (7)), C_2_H_3_ (channel (8)), C_2_H_3_ (channel (9)), ^3^CHCH_3_/C_2_H_4_ (channels (10)), and CO (channels (3)), respectively.

### 4.5 Product Angular and Translational Energy Distribution in the CM Frame

As shown in the Figures 12 and 13 the derived best-fit CM angular distribution, *T*(θ), of all detected primary products was found to be *backward-forward* symmetric with a varying degree of polarization (depending on the product). Therefore, the intensity distribution in the whole CM 0°–180° angular range indicates that these reactive channels occur through a *long-lived complex* mechanism, which implies that the corresponding decomposing intermediate lifetime is longer than 5–6 rotational periods.^69,70,85–87^ For instance, the atomic hydrogen displacement channel (1) is characterized by an isotropic distribution of the C_4_H_5_O product in the whole angular range as described by its best-fit *T*(θ) (Figure 12(*lhs*)-top panel), while the peaking at 25 kJ/mol of the translational energy distribution, *P*(*E*′_T_), for this product channel (Figure 12(*rhs*)-top panel) suggests that this reaction pathway exhibits some exit barrier effect^87^ (see Section 6).

**Figure 12:**
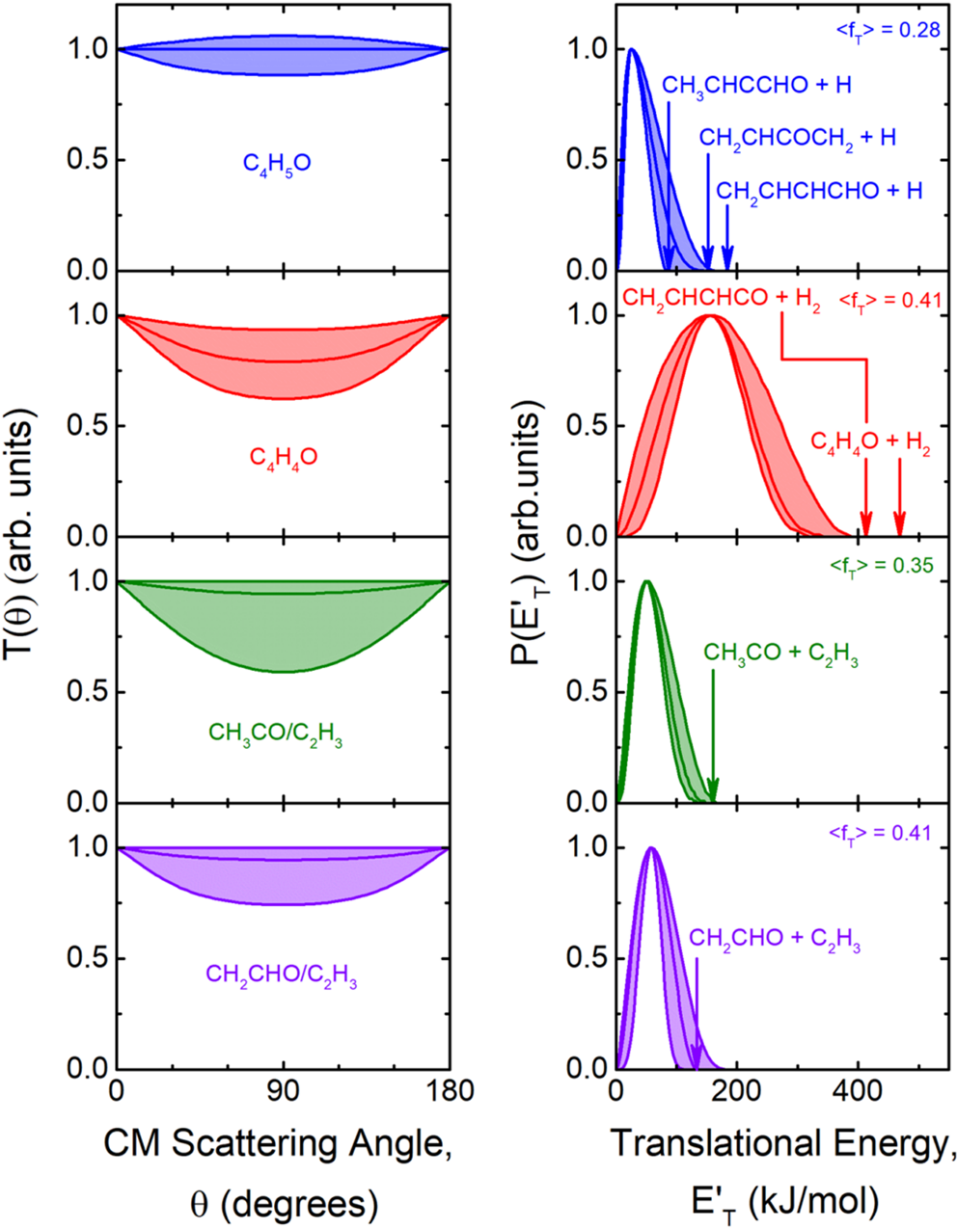
(*lhs*): Best-fit CM angular distributions determined for the C_4_H_5_O (blue), C_4_H_4_O (red), CH_3_CO (green), CH_2_CHO (violet) and CH_2_CO (orange) products. (*rhs*): Best-fit CM translational energy distributions determined for channels (1a,c,f), (2a,b), (8), and (9), whose total available energy (*E*_TOT_) (given by *E*_c_ – ΔH_0_^0^) and average translational energy fractions (⟨*f*_T_⟩) are also shown. The shaded areas represent the error bounds of the derived best-fit CM functions.

**Figure 13:**
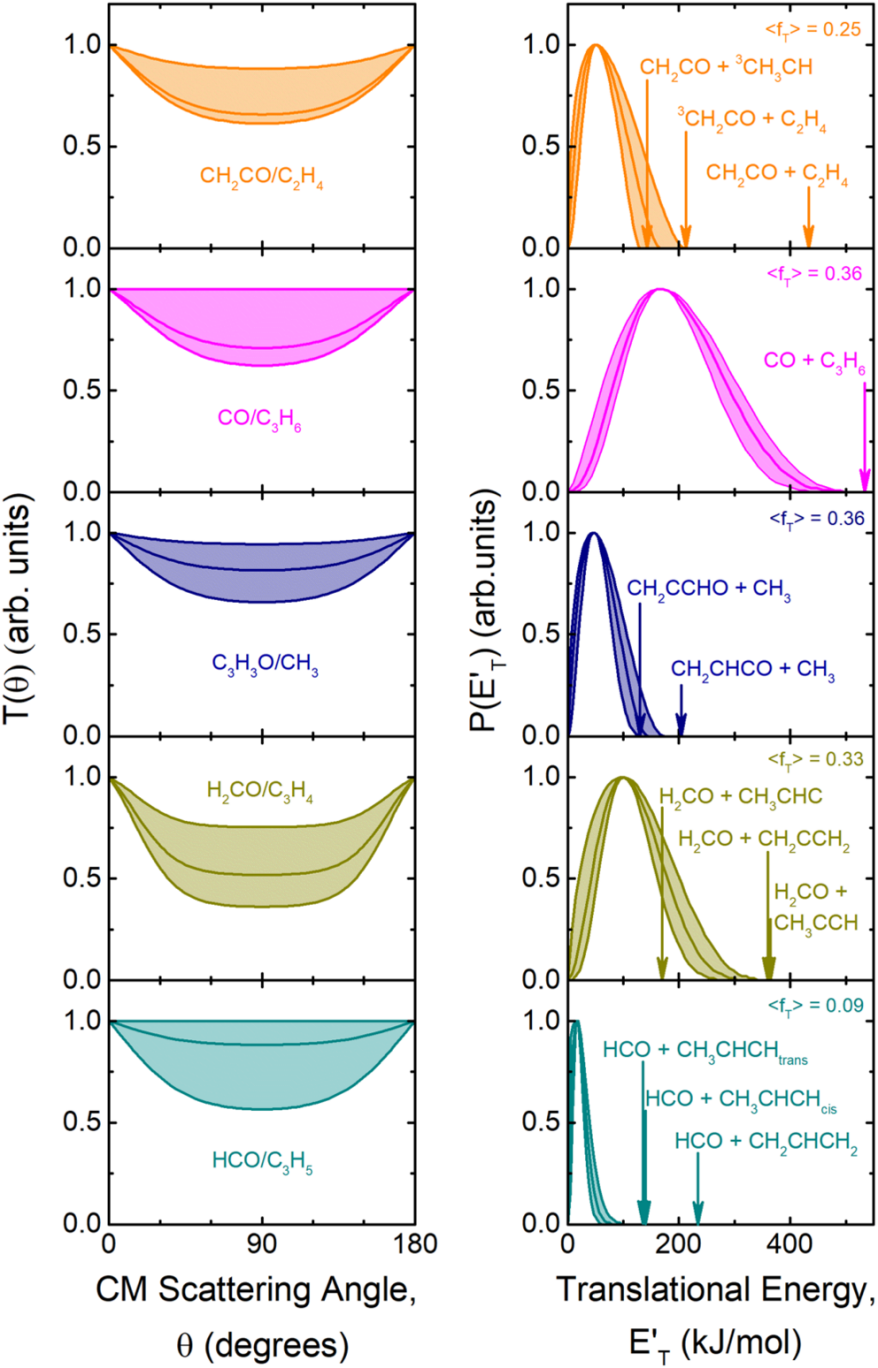
(*lhs*): Best-fit CM angular distributions determined for the indicated CH_2_CO (orange), C_3_H_6_ (magenta), CH_3_ (black), H_2_CO (navy), and HCO (cyan) products. (*rhs*): Best-fit CM translational energy distributions determined for channels (10a–c), (3c), (4a,b), (5a–c), and (7b,c), whose total energy and translational energy fraction are also shown. The shaded areas represent the error bounds of the derived best-fit CM functions.

The *T*(θ) of the C_4_H_4_O coproduct from the H_2_ elimination channel is instead slightly polarized; in addition, the corresponding *P*(*E*′_T_) peaks at very high energy (157 kJ/mol), and this, being the average product translational energy 153 kJ/mol (assuming the open chain isomer coproduct (channel (2b)–see Section 6) and the total available energy for channel (2b) of 370 kJ/mol, gives ⟨*f*_T_⟩ = 0.41 (see Table 1), which indicates the presence of a very high exit barrier. According to the PES the H_2_ channel can only occur via ISC on the singlet PES. Analogously to the H_2_ channel, the peaking at about 170 kJ/mol of the *P*(*E*′_T_) function for the CO + C_3_H_6_ channel (3c) (also this channel can only occur via ISC on the singlet PES) corresponding to an average product translational energy of about 196 kJ/mol (see Table 1), gives ⟨*f*_T_⟩ = 0.36 and indicates that this nonadiabatic channel is characterized by the presence of a high exit barrier on the singlet PES. The presence of very high exit barriers for the H_2_ and CO forming channels and of more modest exit barriers for the H forming channels is supported by the theoretical calculations of the triplet/singlet PESs (see Sections 5 and 6).

**Table 1:** Average Product Translational Energy Fraction, ⟨*f*_T_⟩, and the Total Available Energy, *E*_TOT_, is Listed for Each Characterized Primary Product Channel^a^

product channel	channel number	⟨*f*_T_⟩	⟨*E*_T_⟩ (kJ/mol)	*E*_TOT_ (kJ/mol)
CH_3_CHCCHO + H	1a	0.35	29.8	85.4
CH_2_CHCOCH_2_ + H	1c	0.28	43.0	151.9
CH_2_CHCHCO + H_2_	2b	0.41	152.7	370.0
C_3_H_6_ + CO	3c	0.36	195.8	544.9
HCO + CH_3_CHCH	7b	0.09	22.5	238.5
H_2_CO + CH_3_CCH	5b	0.33	118.5	362.3
CH_2_CHO + C_2_H_3_	9	0.41	56.2	137.2
^3^CH_2_CO + C_2_H_4_	10b	0.25	52.3	211.5
CH_2_CCHO + CH_3_	4a	0.36	52.3	146.4
CH_3_CO + C_2_H_3_	8	0.35	59.5	172.4

a The average product translational energy, ⟨*E*′_T_⟩, can be derived from the equation ⟨*f*_T_⟩ = ⟨*E*′_T_⟩/*E*_TOT_.

As shown in Table 1, all product channels exhibit relatively high ⟨*f*_T_⟩ values, except the HCO + C_3_H_5_ radical channel for which the *P*(*E*′_T_) peaks very close to zero and ⟨*f*_T_⟩ = 0.09, indicating that the dissociation to two radical coproducts of the corresponding bound intermediate occurs without an exit barrier in the exit channel, as it is expected on the singlet PES for radical + radical formation. Correspondingly, the radical coproducts HCO and C_3_H_5_ are expected to be internally (ro-vibrationally) very excited (see Section 6).

### 4.6 Product Branching Fractions

The overall branching fractions of the various detected product channels have been estimated using the procedure implemented by Schmoltner et al.^71^ and that we have widely used in numerous previous CMB studies of multichannel reactions.^28–34,59–63^ The experimental product BF for each of the nine detected isomer product channels are reported in Table 2, with uncertainties ranging from ±20% to ±50% (depending on the channel). As shown in Table 2 experimentally the O­(^3^P) + 1,2-butadiene reaction mainly leads to the CO + C_3_H_6_(propene) channel (3c) with BF = 47.0 ± 12.0%, which results to be the dominant product channel. The second most important channel is that leading to CH_2_CO/^3^CH_2_CO (ketene) + ^3^CHCH_3_/C_2_H_4_ (BF = 24.0 ± 6.0%) (channels (10a,b)), followed by the CH_3_(methyl) + C_3_H_3_O (CH_2_CCHO/CH_2_CHCO) channels (4a,b) (BF = 13.0 ± 3.9%) and H_2_CO­(formaldehyde) + CH_3_CCH/CH_3_CHC channel (5a) (BF = 6.0 ± 1.8%). The other five channels are all found to be minor: formyl radical formation (HCO + CH_3_CHCH) with BF = 3.8 ± 1.5% (channel (7b)); vinoxy radical formation (CH_2_CHO + C_2_H_3_) with BF = 1.2 ± 0.6% (channel (9)); atomic hydrogen displacement (channels (1)) and molecular hydrogen elimination (channels (2)), with BF = 0.8 ± 0.3% and 2.2 ± 0.9%, respectively.

**Table 2:** Product Branching Fractions (BFs) Determined Experimentally (expt) and Theoretically (RRKM/ME) for the O­(^3^P) + 1,2-Butadiene Reaction at *E*_c_ = 41.8 kJ/mol^a^

			RRKM/ME (%)			
products (**T** = triplet PES) (**S** = singlet PES)	channel	expt (%)	total	from **C1** attack	From **C2** attack	From **C3** attack
CH_3_CHCCHO + H (**T**)	1a	**0.8 ± 0.3**	**16.90**	0.63	0	0
CH_2_CHCHCHO + H (**S**)	1f			2.00	0	0
CH_2_CHCOCH_2_ + H (**T**)	1c			1.35	10.77	2.16
C_4_H_4_O + H_2_ (**S**)	2	**2.2 ± 0.9**	**0**	0	0	0
CH_2_CCHO + CH_3_ (**T**)	4a	**13.0 ± 3.9**	**16.77**	0	0	15.02
CH_2_CHCO + CH_3_ (**S**)	4b			0	0	1.75
CH_3_CO + CH_2_CH (**S**)	8	**2.0 ± 0.6**	**1.54**	0	0	1.54
CH_3_CO + CH_2_CH (**T**)				0	0	0
CH_2_CHO + CH_2_CH (**S**)	9	**1.2 ± 0.6**	**0**	0	0	0
CH_2_CO + ^3^CH_3_CH (**T**)	10a	**24.0 ± 6.0**	**19.91**	1.82	5.26	0.99
^3^CH_2_CO + C_2_H_4_ (**T**)	10b			0.55	9.56	1.73
H_2_CO + CH_3_CCH (**S**)	5b	**6.0 ± 1.8**	**4.04**	0	0	0
H_2_CO + CH_3_CHC (**S**)	5a			4.04	0	0
HCO + CH_3_CHCH (**T**)	7a	**3.8 ± 1.5**	**4.40**	0	0	0
HCO + CH_3_CHCH (**S**)	7b			4.40	0	0
HCO + CH_2_CHCH_2_ (**S**)	7c			0	0	0
CO + CH_3_CHCH_2_ (**S**)	3c	**47.0 ± 12.0**	**36.38**	0.82	29.62	5.81
CO + CH_3_CHCH_2_ (**T**)	3a			0.15	0	0

a Also indicated are contributions to the TOTAL theoretical BFs from distinct C1, C2, and C3 attacks, and specific isomeric channels occurring on the triplet and/or singlet PES.

In general, as already mentioned the specific isomeric channels are not distinguishable experimentally. However, this is possible theoretically. The theoretical BFs for specific isomeric channels (from triplet and/or singlet PES), partitioned in the relative contributions of the different attack sites (C1, C2, and C3), are presented in Table 2, along with the theoretical total BFs (obtained by simply adding the partial contributions) that can be compared with the experimental BFs (see Sections 5 and 6).

The analysis of the experimental and theoretical BFs shows that, while some channels can only take place on the singlet PES (such as the CO + propene channel (3c)), other channels can arise from both triplet and singlet PESs. Although from the features of the *P*(*E*′_T_) functions it is possible to infer which isomeric channels are more relevant, for a detailed and quantitative attribution of the various isomeric channels to a given product channel we need to resort to the synergistic contribution from theory. This will be presented in the next Section 5 on theoretical results and discussed in Section 6.

## 5 Theoretical Results

### 5.1 Description of the Triplet and Singlet PESs

Due to the complexity of the system, the PES for the reaction between O­(^3^P) and 1,2-butadiene at 0 K has been divided into three different PESs according to the three possible attack sites for O­(^3^P) on the electrophilic 1,2-butadiene (CH_2_=C=CH–CH_3_), which is characterized by unsaturated bonds covering up to three of the four total carbon atoms. Therefore, O­(^3^P) can either add to C1, representing the terminal carbon of the unsaturated bond system, or to C2 and C3, corresponding to the carbon atom in the middle (C2) and the carbon linked to the methyl group (C3), respectively (see Scheme 1). All the reported energies have been calculated at the CCSD­(T)//ωB97XD/aug-cc-pVTZ level, with relevant energy barriers at the CASPT2 level. The full triplet and singlet PESs for C1, C2, and C3 attack are reported in the Figures S1–S6, respectively, of the Supporting Information (SI). Simplified triplet/singlet PESs, according to the most relevant channels observed and theoretically predicted, with MECPs (where ISC occurs) indicated, are displayed in the Figures 14, 15, and 16 for C1, C2, and C3 attack, respectively (values at CASPT2 level are those in parentheses).

**Figure 14:**
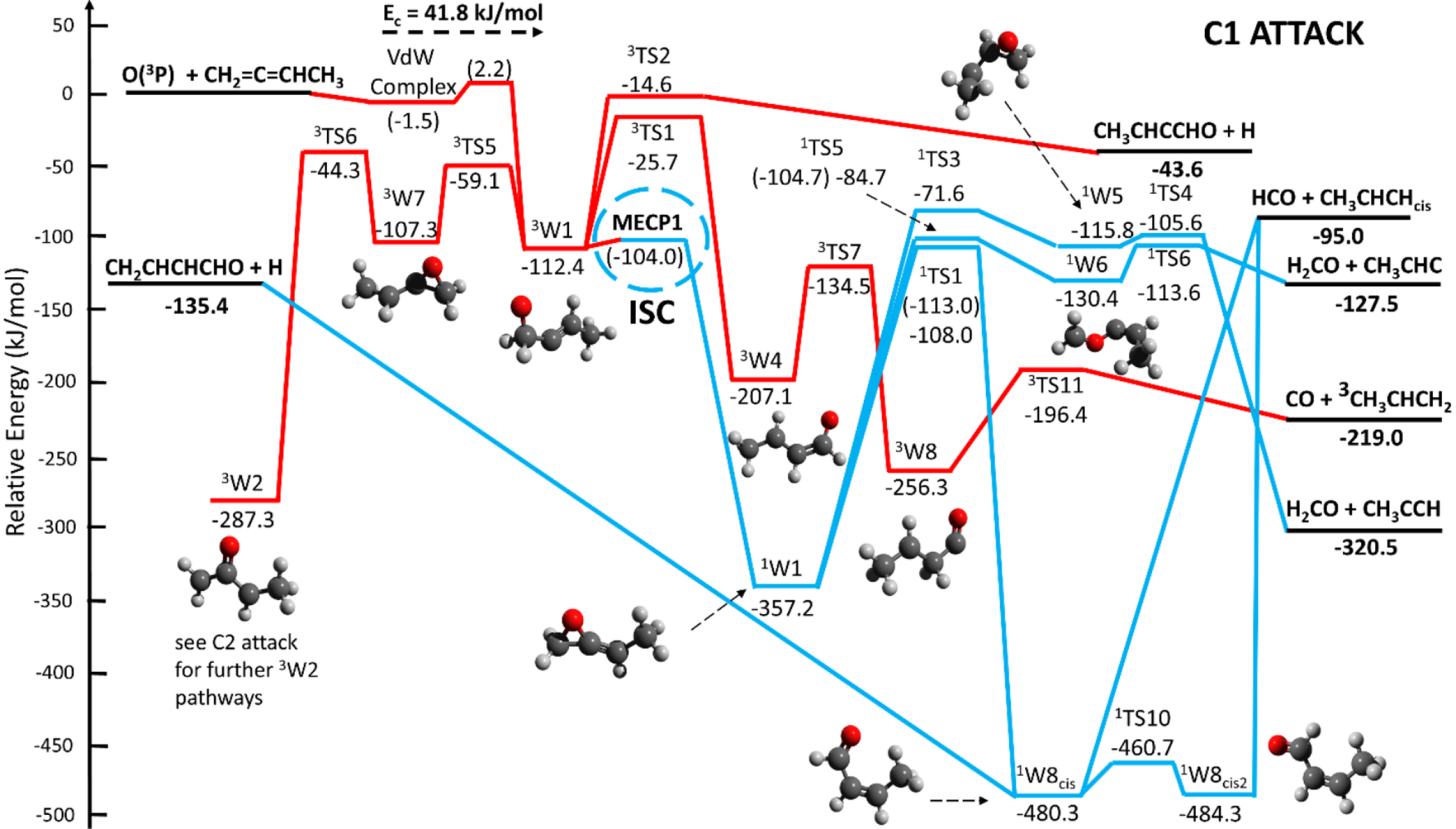
Potential energy surface (simplified; for full PES see triplet and singlet PESs for C1 attack depicted in the Figures S1 and S2) of the O­(^3^P) to 1,2-butadiene system deriving from the electrophilic O­(^3^P) addition to C1. Additional ^1^W1 and ^1^W6 pathways are described in the PES of Figure 15. The PES is calculated at the CCSD­(T)/aug-cc-pVTZ//ωB97XD/aug-cc-pVTZ level of theory, with values in parentheses obtained at the CASPT2 level. The reaction pathways highlighted in bold red and blue solid lines refer to the main reaction pathways accessed by the reactive flux.

**Figure 15:**
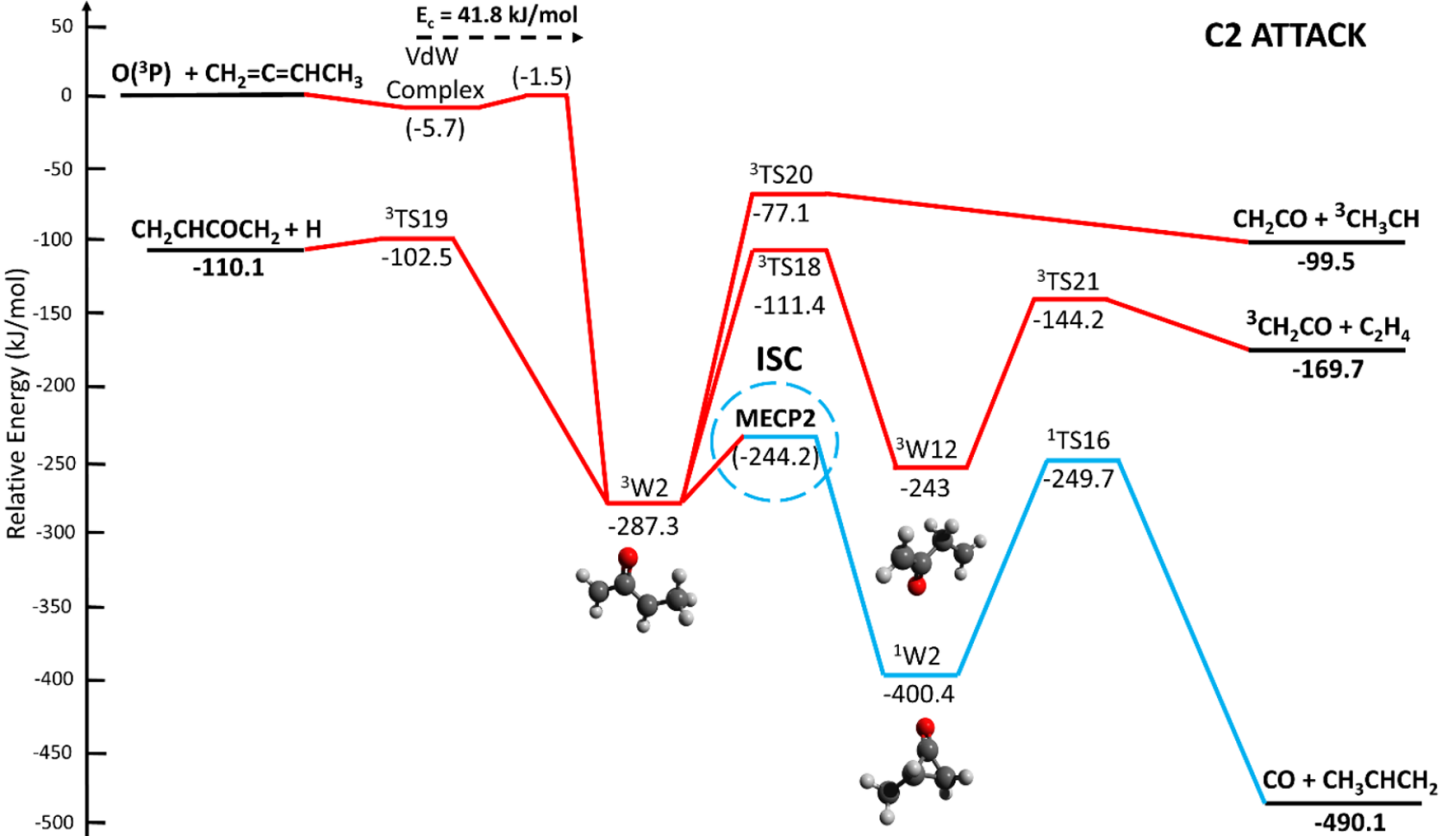
Potential energy surface (simplified; for full PES see triplet and singlet PESs for C2 attack depicted in the Figures S3 and S4) of the O­(^3^P) + 1,2-butadiene system deriving from the electrophilic O­(^3^P) addition to C2. The PES is calculated at the CCSD­(T)/aug-cc-pVTZ//ωB97XD/aug-cc-pVTZ level of theory, with values in parentheses obtained at the CASPT2 level. The reaction pathways highlighted in bold red and blue solid lines refer to the main reaction pathways accessed by the reactive flux.

**Figure 16:**
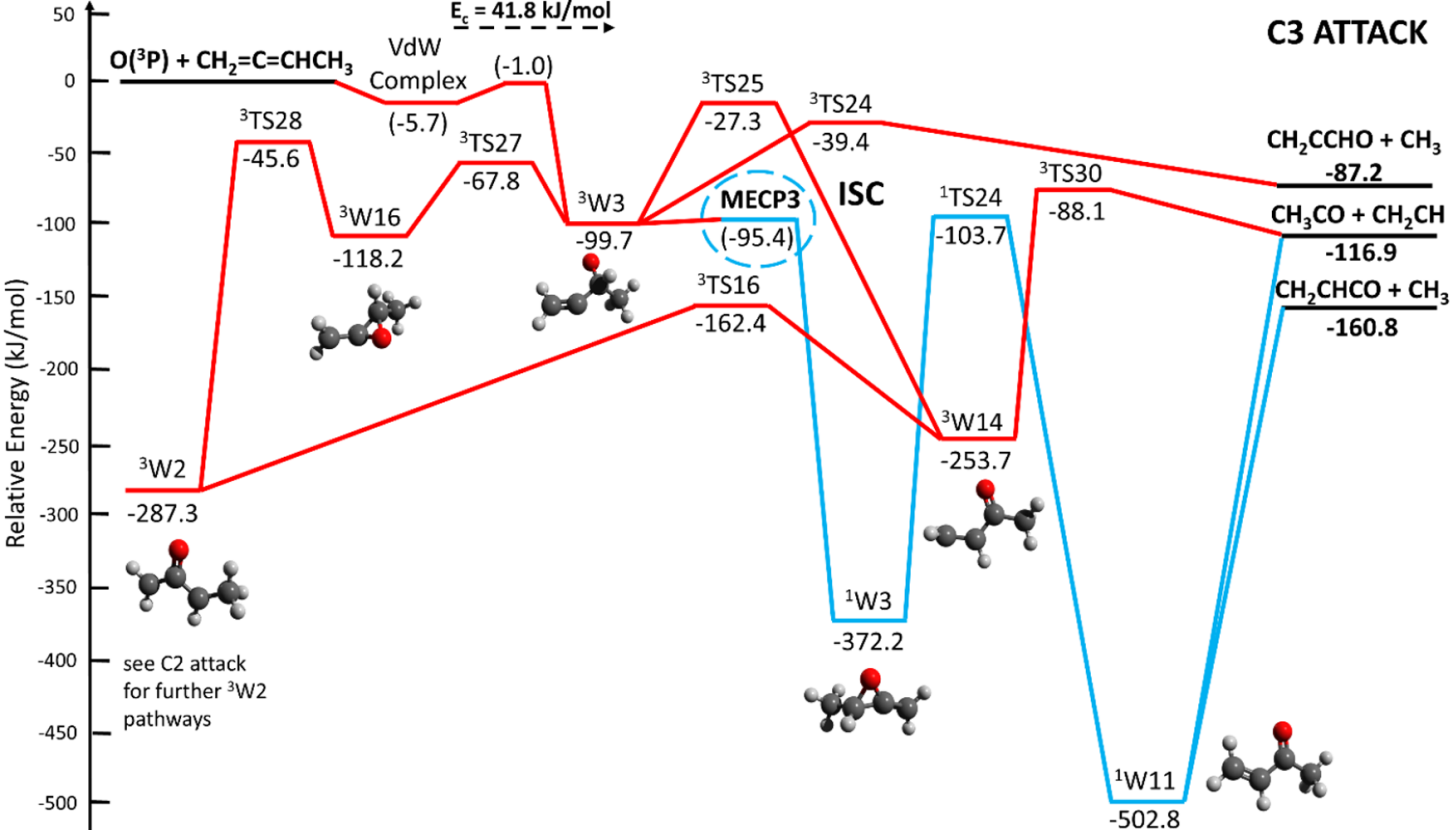
Potential energy surface (simplified; for full PES see triplet and singlet PESs for C3 attack depicted in the Figures S5 and S6) of the O­(^3^P) + 1,2-butadiene system deriving from the electrophilic O­(^3^P) addition to C3. Additional ^3^W2 pathways are described in the potential energy surface of Figure 15. The PES is calculated at the CCSD­(T)/aug-cc-pVTZ//ωB97XD/aug-cc-pVTZ level of theory, with values in parentheses obtained at the CASPT2 level. The reaction pathways highlighted in bold red and blue solid lines refer to the main reaction pathways accessed by the reactive flux.

#### 5.1.1 C1 Attack

Figure 14 illustrates the simplified PES arising from the electrophilic O­(^3^P) addition to C1, which is characterized by a small entrance barrier of 2.2 kJ/mol (calculated at the CASPT2/aug-cc-pVTZ level of theory). The addition reaction results into a high-lying triplet bound intermediate (^3^W1) located at −112.4 kJ/mol, which can competitively (i) dissociate to CH_3_CHCCHO + H (channel 1a) by overcoming a barrier of 97.8 kJ/mol from the bottom of the ^3^W1 well (exit barrier of 29 kJ/mol with respect to products), (ii) isomerize to ^3^W4 by overcoming a barrier (^3^TS1) of 86.7 kJ/mol (from bottom of ^3^W1), (iii) isomerize via ^3^TS5 (−59.1 kJ/mol) to ^3^W7 (−107.3 kJ/mol) which can further isomerize via ^3^TS6 (−44.3 kJ/mol) to ^3^W2 (−287.3 kJ/mol) (for the sake of simplicity, the dissociation pathways of ^3^W2 are illustrated in the PES of Figure 15 and discussed in the next paragraph), and (iv) undergo ISC (at MECP1 located at −104.0 kJ/mol) to the strongly bound ^1^W1 intermediate (located at −357.2 kJ/mol). While ^3^W4 (located at −207.1 kJ/mol), after isomerization to ^3^W8 (located at −256.3 kJ/mol) leads, via ^3^TS11 (−196.4 kJ/mol), to the products CO + ^3^CH_3_CHCH_2_ (triplet propene) (channel 3a), the singlet intermediate ^1^W1 can competitively (i) isomerize via ^1^TS1 (−113.0 kJ/mol) to the very stable ^1^W8_cis_ (−480.3 kJ/mol) that, in turn, can lead directly through a barrierless pathway, or via further facile isomerization to ^1^W8_cis2_, to HCO + CH_3_CHCH_*cis*_ (channel 7b), (ii) isomerize via ^1^TS5 (−104.7 kJ/mol) to ^1^W6 (−130.4 kJ/mol) that dissociates to the products H_2_CO + CH_3_CHC (channel 5a) via a low exit barrier ^1^TS6 (of about 14 kJ/mol with respect to products), and (iii) isomerize to ^1^W5 via ^1^TS3 (at −71.6 kJ/mol) that can readily dissociate to the strongly exothermic H_2_CO + CH_3_CCH (propyne) channel (5b) via ^1^TS4 (however, this pathway is expected to be unfavored with respect to pathway (ii) because ^1^TS3 is substantially higher than ^1^TS5).

#### 5.1.2 C2 Attack

Figure 15 illustrates the simplified PES arising from the electrophilic O­(^3^P) addition to C2, characterized by a submerged entrance barrier of −1.5 kJ/mol calculated at the CASPT2/aug-cc- pVTZ level of theory. The addition reaction results into a low-lying triplet bound (resonance stabilized) intermediate (^3^W2) located at −287.3 kJ/mol, which can competitively (i) dissociate to CH_2_CO + ^3^CH_3_CH (channel 10a) by overpassing a sizable barrier of 210.2 kJ/mol (from the ^3^W2 well) (with an exit barrier of 22.4 kJ/mol with respect to products), (ii) isomerize to ^3^W12 (located at −243.0 kJ/mol) via ^3^TS18 (at −111.4 kJ/mol), that can lead to ketene ^3^CH_2_CO + C_2_H_4_ (channel 10b) by overcoming a modest barrier of 98.8 kJ/mol (from the ^3^W12 well), with an exit barrier of 25.5 kJ/mol with respect to products, and (iii) undergo ISC at MECP2 (−244.2 kJ/mol) to ^1^W2 that leads to CO + CH_3_CHCH_2_ (propene) (channel 3c) through ^1^TS16 (at −249.7 kJ/mol) with a very high exit barrier of 240.4 kJ/mol with respect to products. In competition with the latter pathway, ^1^W2 can also isomerize in two steps to ^1^W10 that can lead to the barrierless formation of CH_2_CHO + CH_2_CH (channel (9)) and, through a very high exit barrier (^1^TS22, located at −172.9 kJ/mol) to H_2_ elimination with coproduct CH_2_CHCHCO (vinylketene) (channel (2b)) with an exit barrier of 197.1 kJ/mol with respect to products (see Figure S4 in the SI). However, these pathways are not found to be competitive (BF = 0, see Table 2) with respect to ^1^W2 dissociation to CO + CH_3_CHCH_2_ (channel 3c) via ^1^TS16.

#### 5.1.3 C3 Attack

Figure 16 shows the simplified PES deriving from the electrophilic O­(^3^P) addition to C3, which is characterized by a submerged barrier of −1.0 kJ/mol calculated at the CASPT2/aug-cc-pVTZ level of theory. The addition reaction gives a high-lying triplet bound intermediate (^3^W3) located at −99.7 kJ/mol, from which a CH_3_ ejection can originate with products CH_2_CCHO + CH_3_ (channel 4a) via ^3^TS24 (at −39.4 kJ/mol) and an exit barrier of 47.8 kJ/mol with respect to products. Competitively, ^3^W3 can isomerize (i) to ^3^W14 (at −253.7 kJ/mol) via ^3^TS25 (at −27.3 kJ/mol) that can lead to CH_3_CO + CH_2_CH (channel 8) with an exit barrier of 28.8 kJ/mol with respect to products, and, competitively, to ^3^W16 (at −118.2 kJ/mol) via ^3^TS27 (at −67.8 kJ/mol) (leading via further isomerization to ^3^W2 (at −287.3 kJ/mol) through ^3^TS28 (at −45.6 kJ/mol) (see Figure 15 for further evolution and fate of ^3^W2). Additionally, ^3^W3 can undergo ISC at MECP3 (located at −95.4 kJ/mol) to ^1^W3 (at −372.2 kJ/mol) that can isomerize to ^1^W11 (at −502.8 kJ/mol, the most stable intermediate in all PES) through ^1^TS24 (at −103.7 kJ/mol) that in turn leads competitively and barrierless to CH_2_CHCO + CH_3_ (channel 4b) and to the somewhat less exothermic CH_3_CO + CH_2_CH channel (8).

### 5.2 Total and Channel-Specific Addition Rates

Total and channel-specific addition rate constants of O­(^3^P) to 1,2 butadiene were computed using VTST at different levels of theory. At the highest, energies and structures were determined at the CASPT2/aug-cc-pVTZ level, with Hessians calculated at the ωB97X-D/aug-cc-pVTZ level on structures determined at the same level of theory. The energy barriers so determined are 3.0, −1.7 and −0.3 kJ/mol for addition to the C1, C2, and C3 sites, respectively (not indicated in the Figures 14–16). The energy barriers are only slightly affected by the level at which structures were optimized, as CASPT2//ωB97X-D energy barriers are 2.2, −1.5, and −1.0 kJ/mol, respectively (indicated in the Figures 14–16). Two different van der Waals (vdW) wells were found, one that is shared for C2 and C3 addition, and the other for C1 addition. The CASPT2//ωB97X-D ZPE corrected energy of the van der Waals well for addition to C2 and C3, which is connected to both saddle points, is −5.7 kJ/mol (see Figures 15 and 16), while that for C1 addition is shallower, having an energy of −1.5 kJ/mol (see Figure 14). The energy barrier for H abstraction from the methyl group is considerably higher, 23.3 kJ/mol when computed at the CCSD­(T)// ωB97X-D level and 21.4 kJ/mol at the CASPT2//ωB97X-D level, using a (10e,9o) active space and the aug-cc-pVTZ basis set (see Figure S3). The rate constant for H abstraction was multiplied by a factor of 2 since the ground and excited state are almost degenerate, as the energy difference computed using multistate CASPT2 calculations is only 0.75 kJ/mol.

The total and the attack site-specific rate constants calculated for O­(^3^P) addition to 1,2-C_4_H_6_ are reported in Figure 17 as a function of temperature, together with that calculated for H-abstraction from the methyl group. As it can be noticed the agreement with the only available experimental data at 293 K^64^ is quite good, which supports the reliability of the adopted computational protocol for the system under investigation. The BFs calculated at 300 K, are 0.16, 0.55, and 0.29 for addition to the C1, C2, and C3 sites, respectively. It is not surprising that the C2 attack gives the largest contribution to the total reactivity, because the corresponding intermediate ^3^W2 is much more stable than ^3^W1 and ^3^W3 (see Figures 14–16), being ^3^W2 stabilized by resonance (as in the O­(^3^P) + allene case^88^).

**Figure 17:**
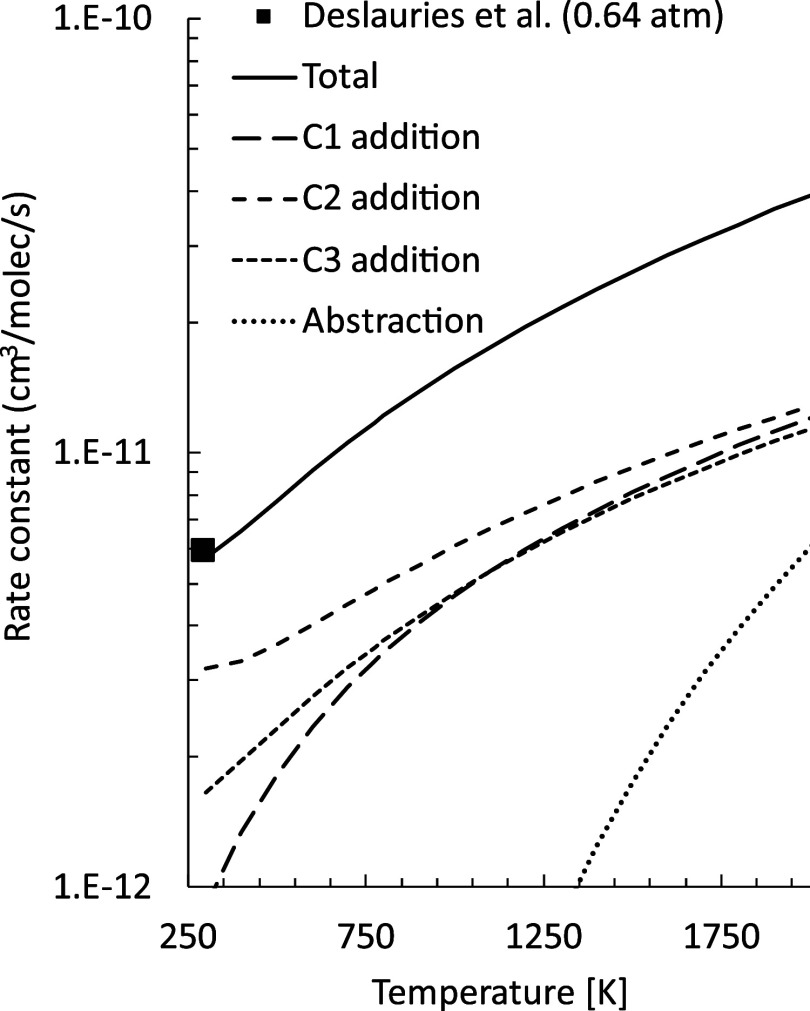
Theoretical total and attack site-specific rate constants for the O­(^3^P) + 1,2-butadiene addition and H-abstraction from methyl reactions as a function of temperature, compared with the experimental data of Deslauries et al.^64^ at 293 K (the experimental uncertainty does not exceed the symbol size).

### 5.3 Master Equation Simulations

The analysis of the stochastic master equation simulations reveals a relatively complicated system reactivity. The summary of the overall reaction fluxes reported in Figure 18, obtained postprocessing the results of the stochastic ME simulations, shows in fact that the reactivity on the triplet PES is not limited to the dissociation reaction pathways directly accessible from the entrance well or to ISC to the singlet PES, but that also isomerization among the entrance wells is possible. In particular a significant portion of the flux entering through addition to C1 or C3 isomerizes to well ^3^W_2_, which is also directly accessed following addition to C2. The reactivity of ^3^W_2_ influences therefore significantly that of the whole system. From ^3^W_2_ it is in fact possible to decompose to H + CH_2_CHCOCH_2_ (channel (1c)), CH_2_CO + ^3^CH_3_CH (channel (10a)), ^3^CH_2_CO + C_2_H_4_ (channel (10b)), or undergo ISC, following which the main reaction pathway is decomposition to CO and propene (channel (3c)). This means that the ^3^W_2_ reactivity accounts for about 70% of the total reactive fluxes, which is split about equally between ISC to the singlet PES and decomposition. It is interesting to notice that the reaction fluxes to CH_2_CO + ^3^CH_3_CH and ^3^CH_2_CO + C_2_H_4_ are similar, with the second channel being about a factor of 1.5 faster (see Table 2), because it is favored by an energy barrier that is about 35 kJ/mol smaller (see Figure 15). The reason why these two channels are competitive is that the first is a β-decomposition reaction while the second an isomerization, followed by β-decomposition. The transition state of the β-decomposition reaction, ^3^TS20, is looser, thus entropically favored with respect to that of the isomerization reaction, ^3^TS18. As a consequence, because of an enthalpy–entropy compensation effect, the two reactions have comparable rates in the considered CMB conditions. For what concerns the reaction channels accessed following C1 and C3 addition the main reaction pathways, in addition to isomerization to ^3^W_2_, are direct decomposition on the triplet PES, which is quantitatively relevant only for the methyl loss channel (4a) coming from ^3^W_3_, or ISC to the singlet PES at MECP3, from which different decomposition barrierless channels are accessible (leading to CH_3_ (channel (4b)) and to CH_3_CO (channel (8))).

**Figure 18:**
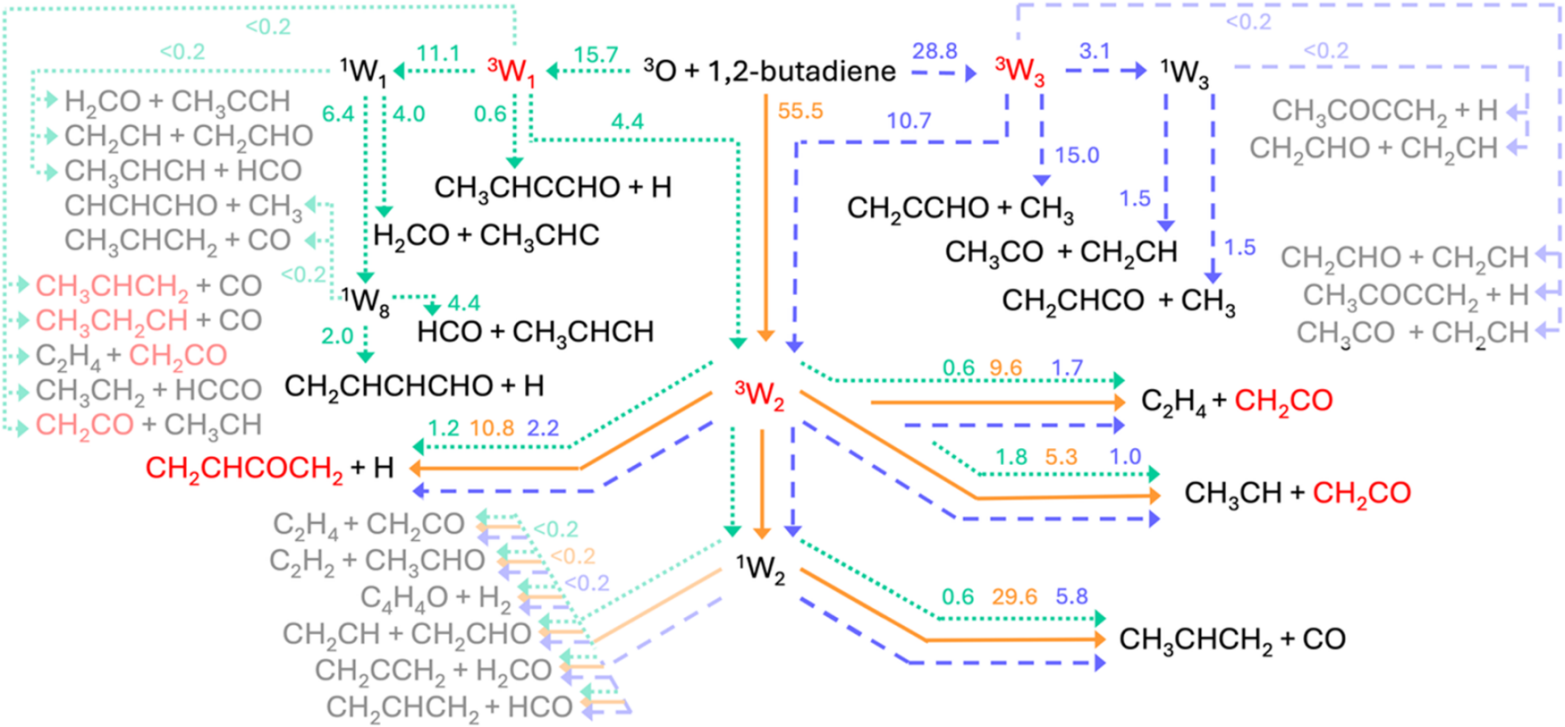
Schematization of the reaction fluxes that follow O­(^3^P) addition to the C1, C2, and C3 sites, leading respectively to the formation of the ^3^W_1_, ^3^W_2_, and ^3^W_3_ entrance wells with 0.157, 0.555, and 0.288 BFs, respectively. The main reaction pathways that are followed after O­(^3^P) addition are connected by bold lines, while the minor pathways considered in the calculations are shaded. The pathways followed after addition to C1, C2, and C3 are highlighted in green, orange, and blue, respectively.

## 6 Discussion on Synergistic Experimental and Theoretical Results

We now discuss our experimental results in the light of the features of the coupled triplet/singlet PESs and related statistical calculations of the product BFs. The dynamics of the O­(^3^P) + 1,2-butadiene reaction derived from the present CMB experiments are contained in the best-fit CM functions (Figures 12 and 13) and product BFs (Table 2) for the nine characterized product channels. In Table 2 the experimental BFs are compared with the theoretical ones; the latter are partitioned in partial contributions (site-specific BFs) from the three possible attack sites C1, C2, and C3 for each isomeric product channel formed on the triplet and/or singlet PES. The theoretical site- and isomer-specific product partial BFs and the total BFs for each global product channel reported in Table 2 are obtained from the partial BF values listed in the flow diagram of Figure 18.

One could expect that the dynamics of the O­(^3^P) + 1,2-butadiene reaction are similar, to some extent, to those previously observed for the related reactions O­(^3^P) + allene,^35^ O­(^3^P) + 1-butene,^36^ and O­(^3^P) + 1,3-butadiene,^28^ albeit these systems differ with respect to the title reaction for the structure of the unsaturated hydrocarbon. In fact, despite the oxidation of cumulenes is characterized by three possible sites for O attack to the unsaturated bonds, in the case of the prototype species, allene, the first and third carbon atom, i.e., the terminal ones, are actually equivalent, while in 1,2-butadiene the third carbon is linked to a methyl group and therefore is not equivalent to the terminal one (see Scheme 1). In addition, although in O­(^3^P) + 1,2-butadiene four carbon atoms make up the hydrocarbon backbone as in 1-butene, in the former case the degree of unsaturation is higher with respect to the corresponding alkene, being similar to 1,3-butadiene, which, however, is not a cumulene but a conjugated diene. It is useful to discuss in detail the dynamics of the various product channels in order to elucidate the overall reaction mechanism. We will exploit the synergy between experimental and theoretical approaches.

### 6.1 H Displacement Channel(s)

As can be seen in Table 2 the kinematically favored (i.e., strongly amplified in the LAB system) H displacement channels (1) are experimentally found to be overall minor (BF = 0.8 ± 0.3%). In contrast, theory overestimate substantially this observation, with the theoretical global BF of the three dominant isomeric H forming channels being 16.90%, a value well outside the experimental error bounds (see Table 2). Although the overall channel (1) is experimentally a minor channel, it is interesting and useful to examine in detail the dynamics of formation of the energetically possible isomeric channels (1a–g) in order to rationalize the combined experimental and theoretical results.

Atomic hydrogen displacement in O­(^3^P) + 1,2-butadiene can energetically occur through different reaction pathways that are determined by the different isomeric intermediates that can originate following the three different attack sites of the atomic oxygen on the unsaturated bonds of the hydrocarbon molecule. These pathways can evolve on both triplet and singlet PESs, and can give rise to a large variety (up to seven) of isomeric H-forming channels (with exothermicity ranging from 43.6 to 142.3 kJ/mol—see Figure 1 and the full PESs in the Figures S1–S6). The probability of formation of the above seven isomeric H-forming channels (1a–g) (listed in Figure 1 in the Introduction in order of increasing exothermicity) is determined by the relative rates of C1, C2, and C3 attacks, and successive relative rates of isomerization and decomposition to products of the relevant intermediates on the coupled triplet/singlet PESs. Specifically, channel (1a) can occur on both triplet and singlet PESs following C1 attack (see Figures S1 and S2); channel (1b) can occur only on the triplet PES following C3 attack (see Figure S5); channel (1c) can only occur on the triplet PES following both C2 (see Figures 15, S3, and S4) and C1 and C3 attack (see Figures 14, 16, S1, S2, S5, and S6, respectively); channel (1d) can only be formed on the singlet PES following C1 attack (see Figure S2); channels (1e) and (1g) can only be formed from ^1^W8_trans_ that can be reached via C2 attack and ISC to ^1^W2 and isomerization to ^1^W8_trans_ (see Figure S4); channel (1f) can only be formed from ^1^W8_cis_ that can be reached from C1 attack and ISC to ^1^W1 and isomerization to ^1^W8_cis_ (see Figure 14).

While the isomer specific BFs on the triplet and singlet PESs can be calculated theoretically through RRMK/ME simulations on the coupled PESs (see Table 2), it is in general difficult to derive detailed information on them experimentally, because the experimental dynamics is much more sensitive to the low energy side and peaking of the *P*(*E*′_T_) function and less to its more energetic side. In general, our technique is not isomer-specific. We recall that the *P*(*E*′_T_) for a specific isomeric channel can extend to a maximum value of energy, *E*_TOT_, given by the sum of the channel exothermicity and the collision energy. It is interesting to note that the cutoff energy of 130 ± 30 kJ/mol of the experimental *P*(*E*′_T_) is essentially consistent with all the isomeric H channels (1a–g). However, in Figure 12(*rhs*)-top panel, indicated with arrows are the only three main channels predicted by theory, namely channels (1a), (1c), and (1f).

The experimental H displacement BF is overall very small (BF = 0.8 ± 0.3%), analogously to what observed for simpler O­(^3^P) + UHs reactions containing at least three C atoms. In fact, at a comparable *E*_c_ the BF is about 1–2% for all systems investigated so far; specifically, it is 2% for O­(^3^P) + propene (C_3_H_6_),^33^ 1.6% for O­(^3^P) + allene (C_3_H_4_),^35^ 1.3% for O­(^3^P) + 1-butene (C_4_H_8_),^36^ while it is significantly larger, 6.2%, for the O­(^3^P) reaction with the conjugated diene 1,3-butadiene (C_4_H_6_).^28^ For the title reaction, theoretically the main contribution to H displacement comes from C2 attack of the O­(^3^P) atom that leads to a contribution of 10.77% (see Table 2) on the triplet PES, forming the ketonic isomer of C_4_H_5_O, namely CH_2_CHCOCH_2_ + H (channel (1c)) (see Figure 15). It should be noted that the H is displaced from the methyl group of 1,2-butadiene. As the schematic PES for C2 attack depicted in Figure 15 shows, the initial triplet diradical intermediate ^3^W2, which lies very low in energy (at −287.3 kJ/mol) being resonance stabilized, can dissociate adiabatically on the triplet PES to three different channels: (i) to CH_2_CHCOCH_2_ + H (channel (1c)) (BF = 10.77%) via a somewhat loose transition state, ^3^TS19 (located at −102.5 kJ/mol), with a relatively small exit barrier (with respect to products) of 7.6 kJ/mol; (ii) to triplet ketene ^3^CH_2_CO + C_2_H_4_ (channel (10b)) (BF = 9.56%) by overpassing the isomerization barrier ^3^TS18 (at −111.4 kJ/mol) to intermediate ^3^W12 (at −243 kJ/mol) that decomposes to products via ^3^TS21 (at −144.2 kJ/mol) with a relatively high exit barrier of 25.5 kJ/mol with respect to products; (iii) to ground state ketene CH_2_CO + ^3^CH_3_CH (triplet ethylidene) (BF = 5.26%) via ^3^TS20 (at −77.1 kJ/mol) with an exit barrier 22.4 kJ/mol with respect to products. In competition to the above three different triplet pathways to products, ^3^W2 can also undergo ISC at MECP2 to ^1^W2 (located at −400.4 kJ/mol) which then decomposes to the most exothermic (Δ*H*_0_^0^ = −490.1 kJ/mol) of all channels, namely CO + CH_3_CHCH_2_ (propene) (channel (3c), BF = 29.62%) located −490.1 kJ/mol via a very high exit barrier (^1^TS16, located at −249.7 kJ/mol) of 240.4 kJ/mol with respect to products. Overall, 55% of the reactive flux of the title reaction proceed following C2 attack (see Table 2 and Figure 15), with slightly above one-half of it via ISC. Clearly, the rate of ISC at MECP2 is critical in determining the main fraction (10.77%) of the H channel, which represents about 2/3 of the overall H BF of 16.90%. In regard, it should be noted that the rate of ISC at MECP2 is calculated to be significantly lower than at MECP1 and MECP3, mainly because the SOC (spin–orbit coupling) at MECP2 is only 9 cm^–1^ against 35 cm^–1^ at both MECP1 and MECP3. Also, while MECP1 and MECP3 are very close in energy to ^3^W1 and ^3^W3, respectively, MECP2 is considerably higher in energy than ^3^W2 (see Figures 14–16).

Table 2 shows that small fractions of the isomeric channel (1c) arise from also C1 attack (BF = 1.35%) and C3 attack (BF = 2.16), because the corresponding initial triplet intermediates ^3^W1 (Figure 14) and ^3^W3 (Figure 16), respectively, can isomerize to ^3^W2, which then leads to channel (1c) through the same pathways described above. Small fractions of other isomeric H channels are also coming from C1 attack, specifically (i) channel (1a) (CH_3_CHCCHO + H) (BF = 0.63%, see Table 2) that occurs adiabatically on the triplet PES (see Figure 14) from the decomposition of the initially intermediate ^3^W1 (located at −112.4 kJ/mol) via ^3^TS2 (located at −14.6 kJ/mol), with an exit barrier with respect to products of 29.0 kJ/mol; (ii) channel (1f) (BF = 2.00%) which is formed on the singlet PES via ISC of ^3^W1 at MECP1 to ^1^W1, which then isomerizes to ^1^W8_cis_ that barrierless decomposes to the strongly exothermic CH_2_CHCHCHO + H channel (1f) (Δ*H*_0_^0^ = −135.4 kJ/mol).

It should be noted that the most exothermic CH_2_CHCHCHO_*trans*_ + H isomer channel (1g) (Δ*H*_0_^0^ = −142.3 kJ/mol), that can only be formed on the singlet PES following C2 attack and ISC from ^3^W2 to ^1^W2 and isomerization to ^1^W8*_trans_* via the very high ^1^TS18 (−133.7 kJ/mol) leading barrierless to CH_2_CHCHCHO + H, results not to be competitive with the C–C bond breaking channel of the ^1^W2 intermediate to the dominant product channel (3c) CO + propene, because this occurs via ^1^TS16 (−249.7 kJ/mol) which is much lower in energy than ^1^TS18 (see Figure S4).

To conclude this analysis, it is important to observe that the theoretical overestimation of the H formation channel with respect to CMB experimental measurements has been systematically observed in our previous studies of reactions between O­(^3^P) and unsaturated hydrocarbons.^28,33,57^ The most reasonable explanation is that the C–H bonds involved in H dissociation reactions are not fully thermalized at the time scale of the reactivity on the triplet PES. This is in contrast with one of the basic assumptions of RRKM theory, the ergodic assumption of statistical energy distribution among all molecular degrees of freedom. Reactions that take place in conditions in which the energy is not statistically distributed are usually referred as non-RRKM reactions, which may thus be the case for the hydrogen loss channels from triplet PES in the CMB systems investigated in this work as well as in our previous studies. Alternative explanations based on uncertainties in the calculated rate constants are less convincing. For example, there is indeed some uncertainty in the ISC crossing rate, and increasing its value would decrease the branching to H. It would though also decrease proportionally the branching to the CH_2_CO channel, for which there is good agreement with experiments. A good agreement between experiment and theory could thus be obtained only if the calculated rate of the H formation channel is decreased substantially. However, the difference between theoretical predictions and experiments for the BF to the H channel is so large, about a factor of 20, that it is unlikely that it can be determined only by the uncertainty in the estimated rate constant for H loss on the triplet PES, which we estimate to be a factor of 2–3.

### 6.2 H_2_ Elimination Channel

We have experimentally observed a small contribution to the total reactivity from also the H_2_ elimination channel (2), with BF = 2.2 ± 0.9%. The *P*(*E*′_T_) for this channel peaks at very high energy (156 kJ/mol) and extends up to the limit of energy conservation for both isomeric channels (2a) and (2b) (see Figure 12(*rhs*) second panel from top). The high ⟨*f*_T_⟩ of 0.41 (assuming channel (1b)–see below) points to a high exit barrier and this is corroborated by the PES calculations. Theoretically, the singlet PES calculations did find the existence of two possible pathways leading to H_2_ elimination, one following C1 attack (see Figure S2), ISC to ^1^W1 and isomerization to ^1^W7 which, via ^1^TS14 (located at −33.9 kJ/mol), can lead to the strongly exothermic furan (C_4_H_4_O) + H_2_ channel (2a) (Δ*H*_0_^0^ = −425. Five kJ/mol). However, because of the very high ^1^TS14 this pathway is statistically predicted not to contribute to H_2_ formation. Another more probable pathway would be that arising from C2 attack (see Figures S3 and S4), ISC to ^1^W2 and two-step isomerization to ^1^W10 that, via ^1^TS22 (located at −172.9 kJ/mol), leads to the H_2_ + CH_2_=CH–CH=CO (vinylketene) channel (2b) (Δ*H*_0_^0^ = −370. 0 kJ/mol) (see Figure S4). Unfortunately, at the present level of the theoretical treatment of the singlet PES, the RRKM/ME calculations did not find appreciable contribution to H_2_ elimination from neither this second energetically open pathway. This is probably due to the simplified form of the singlet PES used in the ME simulations, in which we did not explore in detail the dynamics that follows ISC. In our previous study of O­(^3^P) + propene,^33^ we had in fact found that some H_2_ can be produced via molecular elimination from the diradical complex formed following ISC.

### 6.3 CO + C_3_H_6_ and CH_2_CO/^3^CH_2_CO + ^3^CH_3_CH/C_2_H_4_ Channels

The main primary product channels of the title reaction were found experimentally to be CO + C_3_H_6_(propene) (BF = 47.0 ± 12.0%) (channel 3c) and CH_2_CO + ^3^CH_3_CH and ^3^CH_2_CO + C_2_H_4_ (global BF = 24.0 ± 6.0%) (channels 10a,b) (see Table 2). These experimental BFs agree, within the error bars, with the theoretical total BFs of 36.38% and 19.91%, respectively (Table 2). Notably, the theoretical results permit to disentangle (quantify) the possible pathways leading to CO and ketene formation. Table 2 shows that the O attack on C2 (statistically the favorite attack site, with 49%) contributes the most to the CO channel yield, with a partial BF of 29.62%; however, also pathways originating from C3 attack (BF = 5.81%) and C1 attack (BF = 0.82%) contribute to the overall CO yield on the singlet PES, accessed via ISC to ^1^W2 from ^3^W2, which is reached by isomerization of ^3^W1 and ^3^W3 (see Figures 14–16). Only a minor contribution (BF = 0.15%) to CO formation comes from the triplet PES following C1 attack (see Figure 14). The P­(E′_T_) peaks at a very high energy value (165 kJ/mol) and dies of at 460 ± 40 kJ/mol, almost at the limit of energy conservation, reflecting a high exit barrier. The fraction of energy in product translation is high (⟨f_T_⟩ = 0.36), indicating that the two molecular coproducts are highly internally excited.

The initial intermediate ^3^W2 formed from C2 attack (see Figure 15) can competitively: (i) dissociate directly on the triplet PES, via ^3^TS20, to CH_2_CO + ^3^CH_3_CH products (channel (10a) with an exit barrier of 22.4 kJ/mol (with respect to products), or dissociates via ^3^TS21 to ^3^CH_2_CO + C_2_H_4_ (channel (10b)), following isomerization to ^3^W12, through an exit barrier of 25.5 kJ/mol (with respect to products), and/or (ii) undergo ISC at MECP2 to ^1^W2 that dissociates dominantly to CO + propene via ^1^TS16 located at – 249.7 kJ/mol, that is, with a very high exit barrier of 240.4 kJ/mol with respect to products. For both product channels a *P*(*E*′_T_) peaking far away from zero is expected because of the exit barrier effect, as shown in Figure 13(*rhs*)-top two panels; in fact, the ⟨*f*_T_⟩ is 0.36 and 0.37 for the CO and ketene channel, respectively. Notably, the *P*(*E*′_T_) of the ketene channel dies off at 164 ± 45 kJ/mol, consistent with the exothermicity of both triplet channels (10a) and (10b); this finding rules out the occurrence of ground state ketene + C_2_H_4_ formation on the singlet PES (channel (10c)), despite this channel is strongly exothermic (Δ*H*_0_^0^ = −390.5 kJ/mol) (see Figure S4). The reason is that the intermediate ^1^W2 (located at −400.4 kJ/mol), reached via ISC following C2 attack, does dissociate to CO + propene (channel 3c) much faster than to CH_2_CO + C_2_H_4_ because the barrier ^1^TS16 to CO + C_3_H_6_ formation is at much lower energy (−249.7 kJ/mol) than that (^1^TS17) to ketene formation, which is at −165.6 kJ/mol) (see Figure S4).

Notably, the ketene forming channels occur exclusively on the triplet PES. In fact, there exist two possible isomeric product channels, one leading to CH_2_CO + ^3^CHCH_3_ (channel (10a)) and ^3^CH_2_CO + C_2_H_4_ (channel (10b)), with the main contribution to the total BF coming from C2 attack (Figure 15), followed by a smaller contribution from C1 (Figure 14) and C3 (Figure 16) attacks. Overall, channel (10b) is theoretically about 50% larger than channel (10a) (see Table 2).

Although channels (3) and (10) originate from the same C2 attack site by the atomic oxygen, their dynamics is very different. The comparison of the best-fit P­(E′_T_) functions for these channels indicates that propene formation occurs via ISC from the triplet to the singlet PES, while ketene is generated adiabatically on the triplet PES, as assessed also by the theoretical calculations. In contrast to Cvetanović, who concluded that O­(^3^P) prefers to react with the terminal (less substituted) carbon of the unsaturated hydrocarbon,^9^ in the title reaction the most favorite site of reaction is the central carbon (C2), analogously to what observed in the O­(^3^P) + allene reaction.^27^ Similarly to what observed in the latter system, in the O + 1,2-butadiene reaction the atomic oxygen mainly attacks, in fact, the central carbon leading to CO formation as main product, a pathway characterized by a very high exit barrier (about 240 kJ/mol). For the oxidation of allene the prototype of cumulenes the counter-fragment of CO is C_2_H_4_ (ethylene), while in O + 1,2-butadiene the heavy coproduct is propene. However, in both reactions the CO channel (the main channel) originates via a nonadiabatic transition (ISC) from the triplet to the singlet PES. In addition, the energy of the methyl oxyallyl intermediate (−287.3 kJ/mol respect to the energy of the reactants) and the exit barrier (≈240 kJ/mol with respect to products) which lead to CO formation are comparable in the two reactions (see Figure 15 for the title reaction and Figure 1 of ref 88 for O­(^3^P) + allene). Similarly to the case of the oxyallyl intermediate in the O­(^3^P) + allene reaction, the large stability of the methyl-oxyallyl radical intermediate comes from resonance effects.^35,88^ This intermediate is also associated with further unimolecular pathways. ^1^W8 can, in fact, dissociate both to CH_3_CHCH_*cis*_ + HCO and CH_2_CHO + CH_2_CH (see Figure S2), whereas ^1^W4 can isomerize to ^1^W1 and more probably to ^1^W6 (see Attack C1 of Figure 14 for further ^1^W1 and ^1^W6 pathways). The latter cyclic intermediate can be identified as 2,3-dihydrofuran. It should be noted that the H_2_ channel can be originated following both C1 and C2 attacks. However, theoretically the BF of H_2_ elimination has been found to be negligible (BF = 0) for all three types of attack, because of very high interconversion barriers leading to the intermediate precursors of both furan + H_2_ and CH_2_CHCHCO + H_2_ products (see Figures S1–S3).

### 6.4 CH_3_ (Methyl) Channel

The methyl elimination channel (4) is the third most important channel with experimental BF = 13.0 ± 3.9, and also this value is corroborated nicely by theory which predicts a global BF = 16.77% that originates, following C3 attack, mainly from the triplet PES (BF = 15.02%) and in minor part (BF = 1.75%) from the singlet PES (see Table 2). As can be seen in the PES of Figure 16, the initial triplet intermediate ^3^W3 can decompose to CH_2_CCHO + CH_3_ (channel (4a)) overpassing the tight transition state ^3^TS24, with an exit barrier with respect to products of 60.1 kJ/mol. As shown in Figure 13(*rhs*)-third panel from top, the *P*(*E*′_T_) peaks at 46 kJ/mol and dies off at 146 ± 25 kJ/mol, consistently with a substantial exit barrier height. The same product channel can also be formed via ISC from ^3^W3 to ^1^W3 that after isomerization to ^1^W11 leads through a barrierless path to products. However, for this pathway we would expect a *P*(*E*′_T_) peaking at a much lower energy. Indeed, theoretically the singlet pathway is predicted to contribute for only about 1/12 of the triplet pathway (see Table 2). So, the theoretical predictions are consistent with the experimental indications.

### 6.5 H_2_CO (Formaldehyde) Channel

The H_2_CO product can be accompanied by two different isomeric coproducts: CH_3_CHC (channel (5a)) and/or CH_3_CCH (propyne) (channel 5b). The experimental BF is 6.0 ± 1.8% and also this value is overall corroborated by theory (BF = 4.04) (see Table 2). Notably, the *P*(*E*′_T_) for this channel peaks at about 100 kJ/mol and extends well beyond the limit of energy conservation for channel (5a) up to nearly the total energy for channel (5b) (see Figure 13(*rhs*)-second panel from top). This shape of the *P*(*E*′_T_) function indicates the occurrence of a very exothermic channel, as (5b) is, and the presence of a large exit barrier. As shown in Figure 14, following C1 attack and ISC from ^3^W1 to ^1^W1, the system evolves toward the less exothermic channel H_2_CO + CH_3_CHC overpassing ^1^TS1 (at −113.0 kJ/mol) to ^1^W6 that decomposes to product via ^1^TS6 located at −113.6 kJ/mol, with an exit barrier (with respect to products) of only 13.9 kJ/mol. This pathway is theoretically predicted to be dominant, with BF = 4.04%. However, ^1^W1 could also isomerize to ^1^W5 via the high energy transition state ^1^TS3 at −71.6 kJ/mol. ^1^W5 can in turn readily dissociate to the very exothermic H_2_CO + propyne channel (5b) with an exit barrier (with respect to products) of 215 kJ/mol. However, theory predicts a negligible BF for this pathway, because ^1^W1 can isomerize to ^1^W6 much faster than to ^1^W5 since the latter has a much higher barrier (located at −71.6 kJ/mol) to be overpassed with respect to the former (barrier located at −113.0 kJ/mol).

Notably, of the two possible C_3_H_4_ isomer coproducts, propyne (channel (5b)) and allene (channel (5c)), the latter is expected to be completely negligible because H_2_CO + allene can only be formed following C1 attack, then ISC to ^1^W1, isomerization to ^1^W7 and dissociation of ^1^W7 to H_2_CO + CH_2_CCH_2_ via a prohibitively high transition state, ^1^TS15, located at −15.8 kJ/mol (see Figure S2).

### 6.6 HCO (Formyl) Channel

The HCO + C_3_H_5_ channel (7) has three possible C_3_H_5_ radical isomers, namely ^3^CH_3_CHCH_trans_ (propenyl) (7a) (from C1 attack, see Figure S1), ^1^CH_3_CHCH_cis_ (propenyl) (7b) (from C1 attack, see Figure 14), and CH_2_CHCH_2_ (allyl) (7c) (from C2 attack, see Figure S4). Experimentally this channel is found to represent the fifth most important channel with BF = 3.8 ± 1.5% (Table 2). Notably, the *P*(*E*′_T_) of this channel peaks at very low energy (16 kJ/mol) and dies off very early, at 80 ± 20 kJ/mol; this indicates that the products are formed from a barrierless dissociation of the relevant intermediate(s), and is typical for formation of two molecular radical coproducts (both on triplet and singlet PESs). The cutoff energy value of the *P*(*E*′_T_) function is much lower than the total available energy for all three possible isomeric channels (7a), (7b), and (7c) (see Figure 13(*rhs*)-bottom panel) and this indicates that the two radical coproducts are highly internally excited. Interestingly, theory predicts that the dominant HCO channel is that having the propenyl_trans_ radical (CH_3_CHCH_trans_) as coproduct (channel (7b)) with BF = 4.40%, formed following C1 initial attack, while the other, similarly exothermic propenyl_cis_ channel (7a) (also from C1 attack and from the triplet PES) and the strongly exothermic allyl radical channel (7c) (from C2 attack and from the singlet PES) have both BF = 0 (see Table 2). The reason that the much more exothermic formation of HCO + allyl is negligible (despite arising from the most probable C2 attack) is again because the ^1^W10 intermediate dissociates much faster to CO + propene than to HCO + allyl because of the much lower exit barrier (see Figure S4). The agreement between the experimental and theoretical BFs for the HCO channel is considered very good (see Table 2).

### 6.7 CH_3_CO (Acetyl) and CH_2_CHO (Vinoxy) Channels

The CH_3_CO + C_2_H_3_(vinyl) channel (8) exhibits a *P*(*E*′_T_) that peaks at 50 kJ/mol and dies off at 148 ± 18 kJ/mol, which agrees, within the error bounds, with the total available energy for this channel, being Δ*H*_0_^0^ = – 116.9 kJ/mol and *E*_c_ = 41.8 kJ/mol (see Figure 12(*rhs*))-3rd panel from top. The experimental BF is 2.0 ± 0.6% and this is nicely corroborated by the theoretical prediction of 1.54% (see Table 2). Channel (8) can be formed on both the triplet and singlet PES following C3 attack (see Figure 16). Notably, theory predicts that CH_3_CO + C_2_H_3_ is formed via ISC from ^3^W3 to ^1^W3 at MECP3, followed by ^1^W3 to ^1^W11 isomerization through ^1^TS24 (located at −103.7 kJ/mol) and ensuing barrierless dissociation to products (see Figure 16). This pathway is statistically found dominant with respect to ^3^W3 isomerization to ^3^W14 through the high ^3^TS25 (located at −27.3 kJ/mol) and ensuing dissociation to product via ^3^TS30 (located at −88.1 kJ/mol with respect to reactants) with an exit barrier of 28.8 kJ/mol (with respect to products) (see Figure 16). Clearly ISC at MECP3 is substantially faster than ^3^W3 isomerization to ^3^W14 because ^3^TS25 is located very high in energy.

The high ⟨f_T_⟩ value of 0.35 would suggest a triplet PES contribution to these channels that, however, is not found in the statistical simulations. One reason for that could again be a non- ergodic energy distribution on the triplet PES, similarly to what mentioned for the H channel.

The isomeric CH_2_CHO­(vinoxy) + C_2_H_3_ channel (9) is somewhat less exothermic than channel (8) (by about 27 kJ/mol). The vinoxy channel is experimentally found to be minor (BF = 1.2 ± 0.6%) and smaller than the acetyl channel (8) (see Table 2). It exhibits a *P*(*E*′_T_) shape similar to that derived for channel (8), with a similarly large average fraction of the total available energy released in product translation (⟨*f*_T_⟩ = 0.41). Again, this would indicate an exit barrier effect, as it would be expected on the triplet PES. However, theory finds the BF of channel (9) to be negligible (BF = 0) on both triplet and singlet PESs (see Table 2). This can be rationalized as follows. As in the case of the acetyl channel, the pathway to vinoxy formation on the triplet PES, following C1 attack, has a similarly high barrier (located at −25.7 kJ/mol) to isomerization of the initial triplet intermediate ^3^W1 to other intermediates leading ultimately to vinoxy + C_2_H_3_ (see Figure S1). Regarding the possible pathway on the singlet PES, while in the case of acetyl (see above) the singlet pathway is found theoretically sizable (BF = 1.54%), in the case of vinoxy, as can be seen in Figure 2, the ^1^W2 intermediate, reached via ISC from ^3^W2 following initial C2 attack, has two dissociation pathway possible: (i) to CO + propene via the relatively low ^1^TS16 (located at −249.7 kJ/mol), and this is the dominant channel (3c) for the title reaction, and (ii) to CH_2_CHO + C_2_H_3_ via isomerization through the prohibitively high ^1^TS18 (located at −133.7 kJ/mol) to ^1^W8_trans_ that could further isomerize through ^1^TS19 to ^1^W10. But the latter pathway is not competitive at all with respect to the facile pathway to CO + propene (channel (3c)) and ^3^CH_2_CO + C_2_H_4_ (channel (10b)).

The fact that theory predicts BF = 0 for the experimentally minor vinoxy channel suggests that it is possible that reaction paths that we could not find despite an extensive PES investigation are missing from those reported in the Figures 14–16 and S1–S6.

Finally, a comment is in order about the OH forming channels (12). Of the two isomeric channels, only channel (12a) could have been expected to be of potential relevance. As already mentioned, experimentally no OH formation was observed and this is supported by the theoretical calculations of the PESs and statistical calculations of the product BFs. In fact, this channel could occur via two different pathways: direct H abstraction from the methyl group of 1,2-butadiene or via decomposition of ^3^W2 following C2 addition (see Figure S3-revised). The theoretical kinetic calculations have determined the channel-specific abstraction rate constant as a function of temperature to be negligible at room temperature and still much lower than the addition rate constants even at 1750 K (see revised Figure 17 in main text). The negligible rate of OH formation is determined by the presence of a very high entrance barrier (of more than 20 kJ/mol–see Figure S3-revised) for direct H abstraction from the methyl group forming OH + CH_2_CHCCH_2_ which makes this channel negligible under our experimental conditions. Note that also formation of channel (12a) on the triplet PES following C2 addition (see Figure S3 revised) is negligible (BF = 0), because the decomposition of the initial triplet diradical adduct ^3^W2 to OH + CH_2_CHCCH_2_ is not competitive with the other channels that can be accessed from ^3^W2, such as decomposition to ^3^CH_2_CO + C_2_H_4_ (channel (10b)), ISC, and decomposition to CH_2_CHCOCH_2_ + H because of the relative size of the relevant interconversion barriers (see Figure S3-revised).

### 6.8 Extent of Intersystem Crossing and Comparisons with the Related O­(^3^P) + Allene, O­(^3^P) + 1-Butene, and O­(^3^P) + 1,3-Butadiene Reactions

Having discussed the dynamics of the nine observed product channels by merging experimental and theoretical results, we now focus on the extent of ISC in the title reaction. Because some of the experimentally observed product channels can be formed on both the triplet and singlet PESs, it is not possible to quantify accurately the extent of ISC from only the experimental product BFs. However, we can do that by adding the statistically predicted BFs of the various isomeric product channels, as reported in Table 2, and obtain the total amount of product channels arising from the singlet PES, which gives the extent of ISC. The theoretical extent of ISC amounts to 50.0%. This value is lower than in the case of the related O­(^3^P) + allene reaction for which ISC was estimated to be ≥90%.^35,88^ The reason that ISC in O­(^3^P) + 1,2-butadiene (methylallene) is substantially lower than in O­(^3^P) + allene is because the competitive CH_3_ elimination channel, which dominantly occurs on the triplet PES, is not present in O­(^3^P) + allene. Furthermore, in the title reaction the CH_2_CO/^3^CH_2_CO + ^3^CHCH_3_/C_2_H_4_ triplet channel with its substantial BF of about 20% is much more important than the corresponding CH_2_CO + ^3^CH_2_ channel (BF = 0.3%) in O­(^3^P) + allene. As a consequence, the BFs of the CO + C_2_H_4_(ethylene) and H_2_CO + C_2_H_2_(acetylene) singlet channels in O­(^3^P) + allene^35^ are both substantially larger than the BFs of the corresponding CO + C_3_H_6_ (propene) and H_2_CO + CH_3_CCH propyne/CH_3_CHC channels in O­(^3^P) + 1,2-butadiene.

In contrast to the allene reaction case,^35^ the extent of ISC (50%) for O­(^3^P) + 1,2-butadiene is more similar, at comparable E_c_, to that found in other 4C unsaturated hydrocarbon reactions, such as O­(^3^P) + 1-butene^36^ and O­(^3^P) + 1,3-butadiene^28^ where ISC is found to be about 50% and 66%, respectively.

It is also interesting to compare the BFs of the main analogous channels observed in O­(^3^P) + 1,2-butadiene with those observed in any of the 4C systems so far investigated. In fact, the present product distribution is different from what was observed in O­(^3^P) + 1-butene and 1,3-butadiene at comparable *E*_c_s. In O­(^3^P) + 1-butene, the CO and the CH_2_CO (ketene) channels are not present, while HCO and H_2_CO formation, although not the main channels, were detected in a substantially larger amount (BF = 17% and 15%, respectively) than in the present system. Notably, the main channels in O­(^3^P) + 1-butene^36^ are vinoxy formation (BF = 34%), which is minor in O­(^3^P) + 1,2-butadiene, and CH_3_ formation (BF = 28%), which is about one-half in O­(^3^P) + 1,2-butadiene. In O­(^3^P) + 1,3-butadiene,^28^ the CO channel (BF = 20%) is less than one-half than in O­(^3^P) + 1,2 butadiene, with the main channel being the HCO channel (BF = 36%). In addition, the H_2_CO channel is three times larger (BF = 19%), while the ketene channel is three times smaller (BF = 7%), while the vinoxy channel is much larger (BF = 9.5%), and the CH_3_ channel is not present.

In summary, although the product distributions are very different in the three cases, which is perhaps not surprising because the three hydrocarbons present a different structure, the product BFs for 1,2-butadiene are to some extent closer to those observed for the reaction of 1-butene than 1,3-butadiene. This can be reasonable, since with two nonconsecutive double bonds, the reactivity of 1,3-butadiene will be more similar to that of a molecule (as 1-butene) with only one terminal unsaturated bond.

## 7 Conclusions

The dynamics of the O­(^3^P) + 1,2-butadiene reaction were investigated using a synergistic experimental and theoretical approach. From CMB experiments at the collision energy of 41.8 kJ/mol we observed a total of nine different competitive reactive channels and determined their BFs. Synergistic *ab initio* transition-state theory-based master equation simulations coupled with nonadiabatic transition-state theory on coupled triplet/singlet PESs were employed to compute product BFs and assist the interpretation of the CMB experimental results. Good agreement is found between theoretical predictions and experimental results. In particular theory, through high-level triplet and singlet PES calculations, taking in account triplet to singlet ISC, and RRKM/ME simulations in CMB conditions has quantified the relative role of the electrophilic O atom addition to the three different carbon sites of the 1,2-butadiene molecule and the relative role of triplet and singlet pathways to products. Overall, the theoretical BFs for the main reaction channels agree with the experimental BFs within the experimental uncertainties. Specifically, the channel leading to CO + C_3_H_6_ (propene) is found to be dominant (BF = 47.0 ± 12.0%) and this is corroborated by theory (BF = 36.38%) which elucidates that this channel occurs mainly via O atom addition on the central carbon C2, followed by ISC, with some contributions also from C1 and C3 attacks through isomerization of the initial triplet intermediates to the same triplet intermediate of the C2 attack. Although there is a significant contribution from also C3 addition and a minor one from C1 addition, this particular behavior (dominant C2 attack) is in contrast with respect to Cvetanović “rules” which state that the O atom favorite site of attack is on the less substituted (terminal) carbon involved in the unsaturated bonds (ref 39 and refs therein). The second most important channel is that leading to ketene (CH_2_CO + ^3^CHCH_3_ and ^3^CH_2_CO + C_2_H_4_) (experimental global BF = 24.0 ± 6.0%; theoretical BF = 19.91%) occurring exclusively on the triplet PES following predominantly C2 attack (with minor contributions from also C1 and C3 attacks). The third most important channel is that leading to CH_3_ elimination (experimental BF = 13.0 ± 3.9%; theoretical BF = 16.77%) occurring mainly on the triplet PES (with coproduct CH_2_CCHO) and to a minor extent on the singlet PES via ISC following exclusively C3 attack (with CH_2_CHCO coproduct). Formaldehyde + C_3_H_4_ is the fourth most important channel (experimental BF = 6.0 ± 1.8%; theoretical BF = 4.04%) and is shown to be formed exclusively via ISC following C1 attack. Similar dynamics (C1 attack and ISC) leads to also formation of HCO + C_3_H_5_ (experimental BF = 3.8 ± 1.5%; theoretical BF = 4.4%) which is the fifth most relevant product channel. Minor channels (experimental BF of the order of 1%–2%) are H displacement, H_2_ elimination, CH_3_CO­(acetyl) formation, and CH_2_CHO­(vinoxy) formation. Theory overestimate substantially the H channel and this is attributed to a non-RRKM behavior for H displacement reactions as observed in numerous related O­(^3^P) reactions with 3C and 4C unsaturated hydrocarbons previously investigated.

The reaction mechanism and product distribution for O­(^3^P) + 1,2-butadiene (methylallene) were compared with those observed, at comparable collision energies, in related C4 unsaturated systems, namely O­(^3^P) + 1-butene and O­(^3^P) + 1,3-butadiene, as well as in the prototype of cumulenes, namely O­(^3^P) + allene. Similarities and differences were discussed in terms of the different structure of the unsaturated hydrocarbons.

Theory shows that numerous primary products for the title reaction derive from the occurrence of ISC, such as CO, HCO, H_2_CO, and CH_3_CO formation, and in minor part also H displacement and CH_3_ elimination. The extent of ISC (theoretically predicted to be 50%) results to be more similar to the case of the O­(^3^P) reactions with the related 4C alkene, 1-butene (BF = 50%) and the 4C conjugated diene, 1,3-butadiene (BF = 66%), while substantially lower than the case of the 3C cumulene, allene (BF > 90%), most notably because of the presence of the CH_3_ adiabatic elimination channel in the case of the title reaction and of the minor (BF = 0.3%)^35^ adiabatic channel to ketene (+^3^CH_2_) in the allene reaction.

The total and attack site-specific rate constants were calculated as a function of temperature (293–2000 K). Good agreement is found with the experimental value at 293 K.

The finding of this work can be useful for kinetic modeling of 1,2-butadiene oxidation and of systems where 1,2-butadiene is an important intermediate, as well as for the understanding of differences in laminar burning velocities of 1,2-butadiene and 1,3-butadiene isomers^27^ and their improved modeling.

## Supplementary Material



## Notes

Schematic representation of the potential energy surface for the O­(^3^P) + CH_2_=C=CH–CH_3_ (1,2-butadiene) reaction, obtained considering additional (less favorable) reaction pathways with respect to those reported in Figures 14–16 of the main text (Figures S1–S6) (PDF)
