# Supplementary material for: Crossed-Beams and Theoretical Studies of the Multichannel Reaction O(3P) + 1,2-Butadiene (Methylallene): Product Branching Fractions and Role of Intersystem Crossing
Source: J Phys Chem A. 2025 Aug 26;129(36):8278–302. doi: 10.1021/acs.jpca.5c03937 (PMC12434676; doi:10.1021/acs.jpca.5c03937)
Supplement: Supplementary file 1 [file jp5c03937_si_001.pdf]

Supporting Information for the paper:

Crossed-Beams and Theoretical Studies of the Multichannel Reaction O(^{3}P) + 1,2-Butadiene (Methylallene): Product Branching Fractions and Role of Intersystem Crossing

Gianmarco Vanuzzo[1], Andrea Giustini[2], Adriana Caracciolo[1], Silvia Tanteri[1], Domenico Stranges[3], Marzio Rosi[2], Piergiorgio Casavecchia[1,4,*], Nadia Balucani[1,*], Maristella Di Teodoro[5], Sarah Nicole Elliott[5], Carlo Cavallotti[5,*]

[1]*Dipartimento di Chimica, Biologia e Biotecnologie, Università degli Studi di Perugia, 06123 Perugia, Italy*
[2]*Dipartimento di Ingegneria Civile e Ambientale, Università degli Studi di Perugia, 06125 Perugia, Italy*
[3]*Dipartimento di Chimica, Università degli Studi La Sapienza, 00185 Roma, Italy*
[4]*Computational Laboratory for Hybrid/Organic Photovoltaics (CLHYO), Istituto CNR di Scienze e Tecnologie Chimiche "Giulio Natta" (CNR-SCITEC), 06123 Perugia, Italy*
[5]*Dipartimento di Chimica, Materiali, Ingegneria Chimica "Giulio Natta", Politecnico Milano, 20131 Milano, Italy*

This file contains:

- **Figure S1**. Schematic representation of the triplet potential energy surface for the O(^{3}P) + CH_2=C=CH-CH_3 (1,2-butadiene) reaction, following C1 attack, obtained considering additional (less favorable) reaction pathways with respect to those reported in Figure 14 of the main text.

- **Figure S2**. Schematic representation of the singlet potential energy surface for the O(^{3}P) + CH_2=C=CH-CH_3 (1,2-butadiene) reaction, following C1 attack, obtained considering additional (less favorable) reaction pathways with respect to those reported in Figure 14 of the main text.

- **Figure S3**: Schematic representation of the triplet potential energy surface for the O(^{3}P) + CH_2=C=CH-CH_3 (1,2-butadiene) reaction, following C2 attack (continuous line) and direct abstraction (dashed line), obtained considering additional (less favorable) reaction pathways with respect to those reported in Figure 15 of the main text.

- **Figure S4**: Schematic representation of the singlet potential energy surface for the O(^{3}P) + CH_2=C=CH-CH_3 (1,2-butadiene) reaction, following C2 attack, obtained considering additional (less favorable) reaction pathways with respect to those reported in Figure 15 of the main text.

- **Figure S5**: Schematic representation of the triplet potential energy surface for the O(^{3}P) + CH_2=C=CH-CH_3 (1,2-butadiene) reaction, following C3 attack, obtained considering additional (less favorable) reaction pathways with respect to those reported in Figure 16 of the main text.

- **Figure S6**. Schematic representation of the singlet potential energy surface for the O(^{3}P) + CH_2=C=CH-CH_3 (1,2-butadiene) reaction, following C3 attack, obtained considering additional (less favorable) reaction pathways with respect to those reported in Figure 16 of the main text.

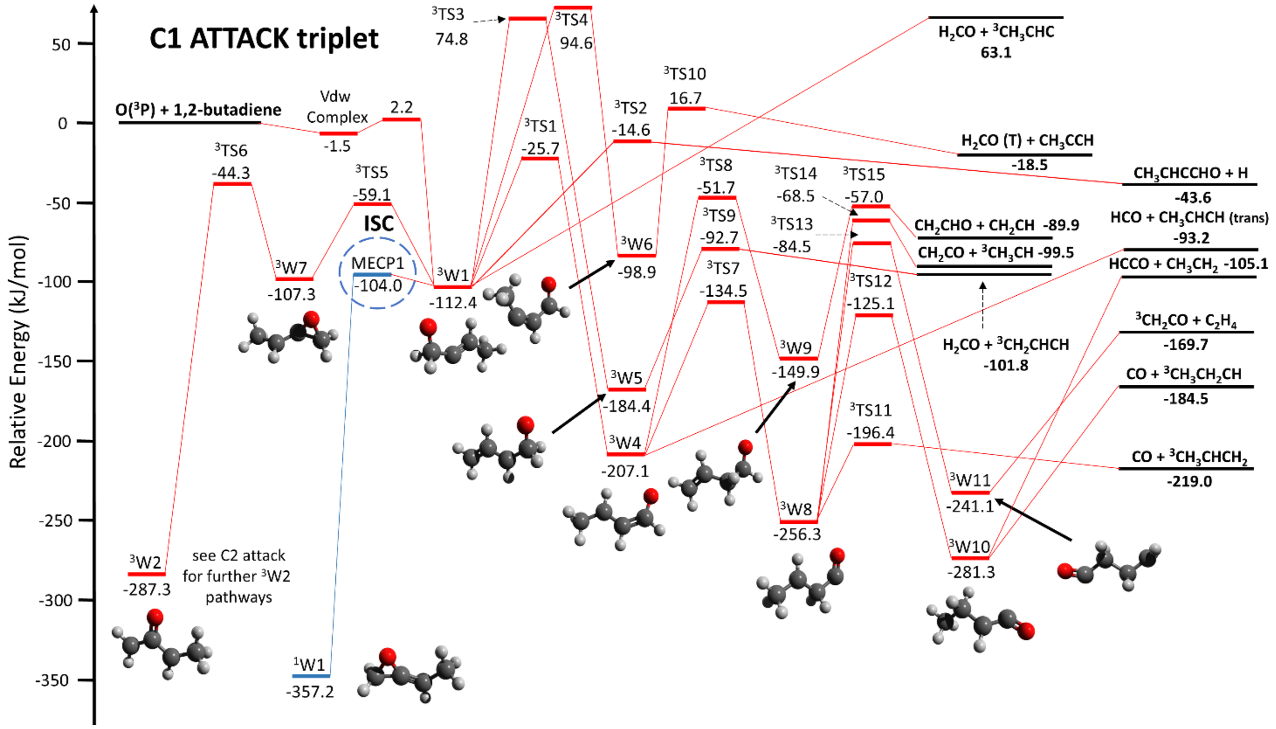

Figure S1

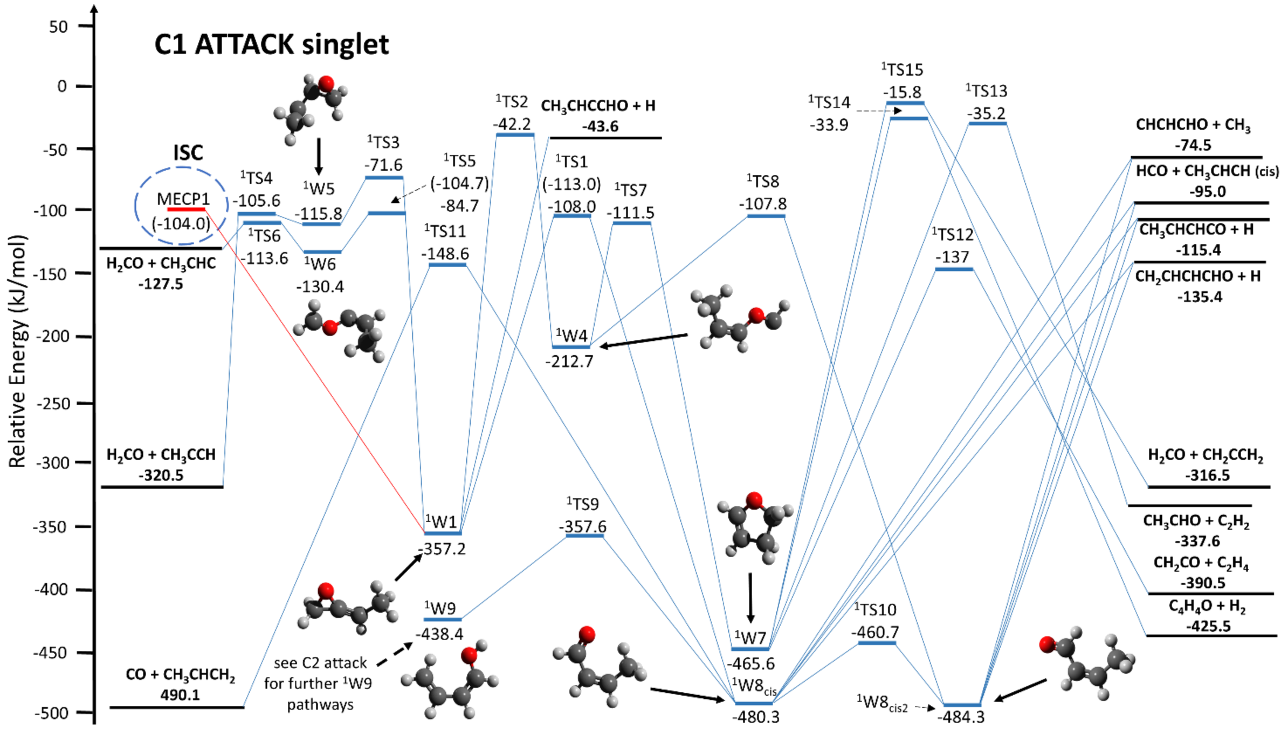

Figure S2

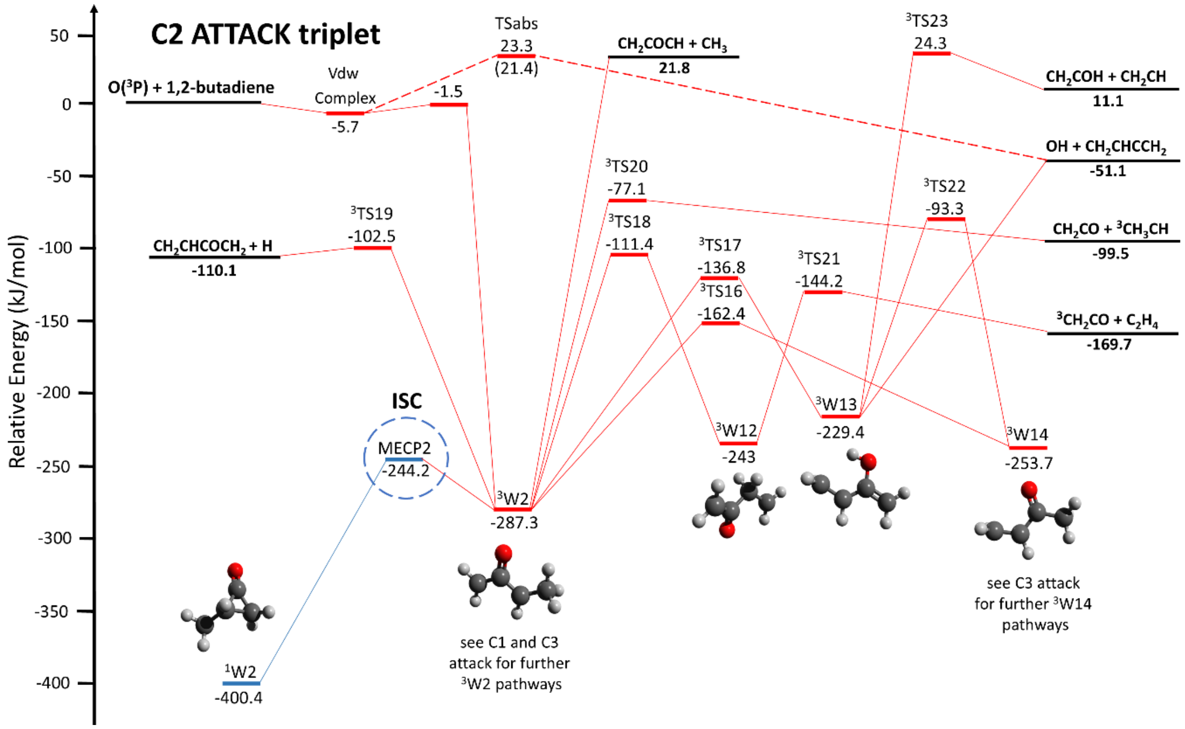

Figure S3

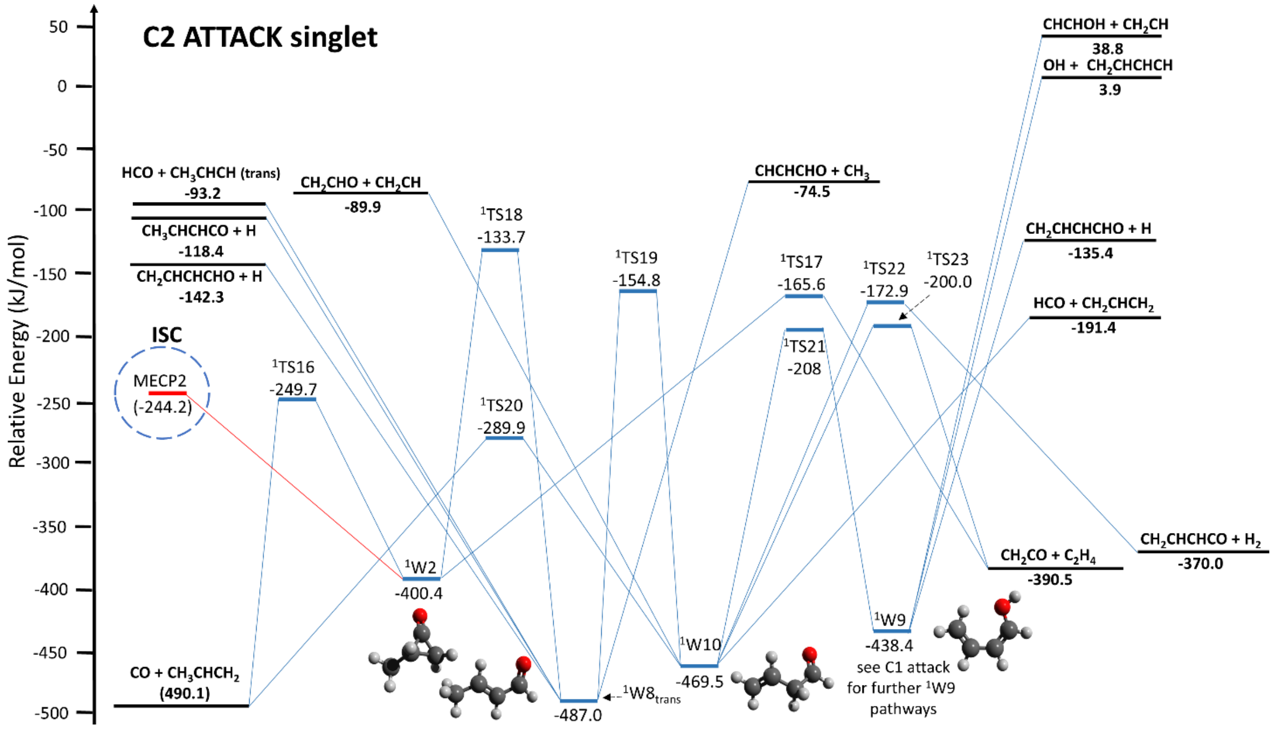

Figure S4

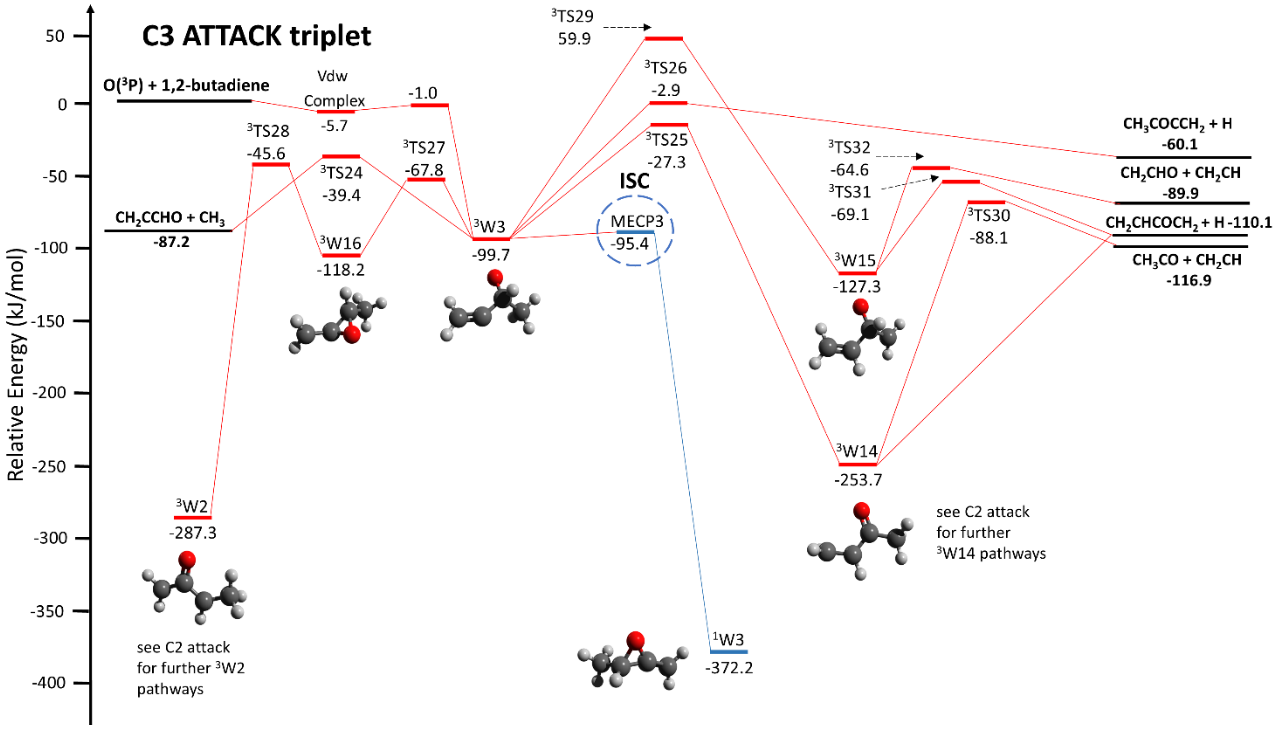

Figure S5

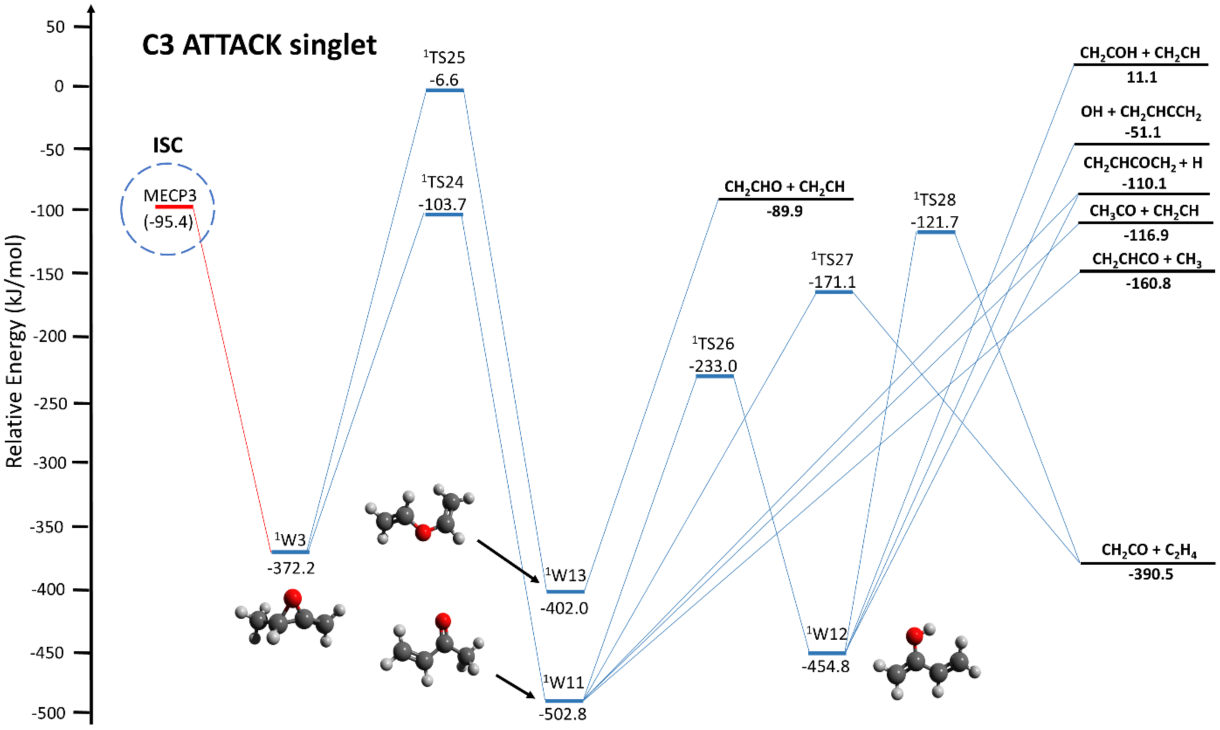

Figure S6
